# Gastric cancer-derived LBP promotes liver metastasis by driving intrahepatic fibrotic pre-metastatic niche formation

**DOI:** 10.1186/s13046-023-02833-8

**Published:** 2023-10-03

**Authors:** Li Xie, Shengkui Qiu, Chen Lu, Chao Gu, Jihuan Wang, Jialun Lv, Lang Fang, Zetian Chen, Ying Li, Tianlu Jiang, Yiwen Xia, Weizhi Wang, Bowen Li, Zekuan Xu

**Affiliations:** 1 Department of General Surgery, The First Affiliated Hospital of Nanjing Medical University, 300 Guangzhou Road, Nanjing, Jiangsu Province 210029 China; 2 Department of General Surgery, Nantong First People’s Hospital, Affiliated Hospital 2 of Nantong University, Nantong, Jiangsu Province 226001 China; 3 Jiangsu Key Lab of Cancer Biomarkers, Prevention and Treatment, Collaborative Innovation Center for Cancer Personalized Medicine, Nanjing Medical University, Nanjing, Jiangsu Province 211166 China

**Keywords:** Gastric cancer, Secreted proteins, LBP, Liver metastasis, TGF-β1, TLR4, Fibrosis, Pre-metastatic niches

## Abstract

**Background:**

Liver metastasis (LM) is one of the most common distant metastases of gastric cancer (GC). However, the mechanisms underlying the LM of GC (GC-LM) remain poorly understood. This study aimed to identify the tumour-secreted protein associated with GC-LM and to investigate the mechanisms by which this secreted protein remodels the liver microenvironment to promote GC-LM.

**Methods:**

Data-independent acquisition mass spectrometry (DIA-MS), mRNA expression microarray, quantitative real-time PCR, enzyme-linked immunosorbent assay (ELISA) and immunohistochemistry (IHC) were performed to identify and validate the GC-secreted proteins associated with GC-LM. A modified intrasplenic injection mouse model of LM was used to evaluate the progression and tumour burden of LM in vivo. Flow cytometry, immunofluorescence (IF), western blots (WB) and IHC were performed to validate the pre-metastatic niche (PMN) formation in the pre-modelling mouse models. mRNA sequencing of PMA-treated THP-1 cells with or without lipopolysaccharide binding protein (LBP) treatment was used to identify the functional target genes of LBP in macrophages. Co-immunoprecipitation (Co-IP), WB, ELISA, IF and Transwell assays were performed to explore the underlying mechanism of LBP in inducing intrahepatic PMN formation.

**Results:**

LBP was identified as a critical secreted protein associated with GC-LM and correlated with a worse prognosis in patients with GC. LBP activated the TLR4/NF-κB pathway to promote TGF-β1 secretion in intrahepatic macrophages, which, in turn, activated hepatic satellite cells (HSCs) to direct intrahepatic fibrotic PMN formation. Additionally, TGF-β1 enhanced the migration and invasion of incoming metastatic GC cells in the liver. Consequently, selective targeting of the TGF-β/Smad signaling pathway with galunisertib demonstrated its efficacy in effectively preventing GC-LM in vivo.

**Conclusions:**

The results of this study provide compelling evidence that serological LBP can serve as a valuable diagnostic biomarker for the early detection of GC-LM. Mechanistically, GC-derived LBP mediates the crosstalk between primary GC cells and the intrahepatic microenvironment by promoting TGF-β1 secretion in intrahepatic macrophages, which induces intrahepatic fibrotic PMN formation to promote GC-LM. Importantly, selectively targeting the TGF-β/Smad signaling pathway with galunisertib represents a promising preventive and therapeutic strategy for GC-LM.

**Supplementary Information:**

The online version contains supplementary material available at 10.1186/s13046-023-02833-8.

## Background

Gastric cancer (GC) is the sixth most prevalent malignant tumour worldwide, and has a high mortality rate [1]. Liver metastasis (LM) represents a major haematologic spread of GC, detected in approximately 4–14% of patients with GC during initial diagnosis [2]. Moreover, it accounts for 37% of postoperative recurrences in patients with GC who have undergone curative gastrectomy [3]. Unfortunately, owing to the delayed detection and limited efficacy of current treatments, the prognosis of patients with GC-LM remains extremely poor, with a 5-year over survival rate of less than 10% [4]. Therefore, it is imperative to further investigate the mechanisms underlying the initial events in GC-LM to identify potential diagnostic markers and effective therapeutic targets for early detection and improved intervention.

Tumour metastasis is a complex process involving multiple steps in which tumour cells spread from the primary sites to foreign organs, ultimately establishing secondary tumours [5]. According to the ‘seed and soil’ hypothesis [6], the communication between tumour cells (the ‘seed’) and the microenvironment of target organs (the ‘soil’) largely determines the efficacy of metastasis [7]. Emerging evidence suggests that primary tumours can initiate metastasis by reshaping the microenvironment of foreign organs through the secretion of soluble factors, including cytokines, extracellular vesicles, or secreted proteins. This process creates permissive and supportive microenvironments that facilitate the colonization and growth of disseminated cancer cells in distant organs [8–11]. These favourable microenvironments for establishing metastases are known as pre-metastatic niches (PMNs) [12]. PMNs are characterized by several features, including extracellular matrix (ECM) reprogramming, immunosuppression, inflammation, angiogenesis/vascular permeability, lymphangiogenesis, organotropism [13]. Increasing evidence has demonstrated that secreted proteins, as major mediators between tumour cells and foreign microenvironments, can promote metastasis by inducing PMNs [14]. However, the role of secreted proteins from GC in GC-LM remains unclear. Therefore, identifying critical secreted proteins that directed intrahepatic PMNs could be a novel strategy to detect GC-LM at an early stage, and investigation of the underlying mechanism may lead to the development of promising therapeutic targets to effectively prevent and control GC-LM.

In the present study, LBP was identified as a critical secreted protein that is associated with GC-LM and corelated with a worse prognosis in patients with GC. LBP, known as an acute-phase protein [15, 16], has been found to be associated with the progression of multiple types of cancer [17, 18]. Several recent bioinformatics studies have reported that LBP level could serve as a prognostic marker in patients with GC, owing to its immune-related role [19–21]. However, the functional roles and underlying mechanisms of LBP in GC-LM remain largely unclear. Here, it was reported that GC-derived LBP drives the formation of intrahepatic fibrotic PMN by inducing TGF-β1 secretion in intrahepatic macrophages to promote GC-LM.

## Methods

### Human tissue and serum samples

IHC staining of LBP and TGFBR1 was performed in the tissues obtained from primary GC tumors (120 cases), para-cancer normal gastric tissues (30 cases), and LM tissues (15 cases) of patients with GC, all of which were collected from the First Affiliated Hospital of Nanjing Medical University between 2010 and 2019. The serum samples from two independent cohorts of patients with GC or healthy volunteers without malignancy or inflammatory disease were collected from the First Affiliated Hospital of Nanjing Medical University between 2015 and 2019, prior to any treatment or surgery. Cohort 1 consisted of 20 healthy volunteers, 30 patients with GC at stage II/III, and 30 patients with GC-LM. Cohort 2 included additional 60 patients with GC at stage II/III. All samples were collected with informed consent from patients or healthy volunteers, and the study was conducted with approval from the ethics committee of Nanjing Medical University, in compliance with the Declaration of Helsinki.

### Microarray analysis

The SurePrint G3 Human mRNA Expression Microarray (4 × 180 k) was utilized to determine the gene expression profile of 24 GC tissues and their paired para-cancer normal gastric tissues, as reported in our previous study [22]. Raw data acquisition was performed using the US Agilent microarray scanner, and data normalization was carried out using the GeneSpring software GX 11.0. The mRNA microarray data are presented in Supplementary Table 1.

### Data-independent acquisition mass spectrometry (DIA-MS)

To determine the secretome profiling of serum from 5 patients with GC-LM and 5 GC patients without LM (GC-NLM) at stage III, after removing high-abundance proteins, DIA-MS was performed on the serum samples using a Thermo Fisher's Q Exactive HF Hybrid Quadrupole OrbitrapTM (QE-HF) mass spectrometer (OE Biotechnology, Shanghai, China) according to the manufacturer's instruments. The secretome profiling data are presented in Supplementary Table 2.

### Cell culture

The human HSCs cell line LX-2 was obtained from Merck (Darmstadt, Germany), while the human GC cell lines (AGS, HGC-27, MKN45, MKN74, SNU-1, NCI-N87, KATOIII), human normal gastric epithelial cell line GES -1 and human leukemia monocytic cell line THP-1 were obtained from the Cell Bank of the Chinese Academy of Sciences. LX-2 cells were grown in DMEM (Merck, Germany) supplemented with 10% FBS (Gibco, USA). AGS cells were grown in F-12 K Medium (Gibco, USA) with 10% FBS, while MKN45, MKN74, SUN-1, HGC-27, and NCI-N87 cells were grown in RPMI-1640 (Gibco, USA) with 10% FBS. KATOIII cells were grown in Iscove's Modified Dulbecco's Medium (IMDM) (Gibco, USA) with 20% FBS. All mediums were supplemented with 100 mg/ml penicillin/streptomycin. Primary mouse HSCs were isolated as previously reported [23] and cultured in DMEM (Gibco, USA) supplemented with 10% FBS and 100 mg/ml penicillin/streptomycin. All cell lines were authenticated by short tandem repeat (STR) profiling and tested to be free from mycoplasma contamination. All cells were cultured in a cell incubator with 5% CO2 at 37 °C under humidified conditions.

### RNA extraction and quantitative PCR

Total RNA was extracted from the cell and tissue samples using TRIzol reagent (Technologies, USA) and a RNeasy Mini kit (Qiagen, Germany) following the manufacturer’s protocol. The mRNA was transcribed into cDNA using the PrimeScript RT Master Mix Kit (TaKaRa, Japan), and the cDNAs were amplified using the FastStart Universal SYBR Green Master Kit (Roche, Mannheim, Germany), according to the manufacturer's protocol. The relative expression of mRNA was normalized with the expression of GAPDH or ACTB mRNA. Primer information is provided in Supplementary Table 3.

### Plasmids and lentivirus transfection

To generate LBP and TLR4 overexpression plasmids, human LBP and TLR4 cDNAs were synthesized separately using PrimerSTAR Max DNA Polymerase Mix (Takara, RR036A, Japan) and subcloned into the pENTR vector fused with 3X-Flag at the C terminus (Vigene Biosciences, Shandong, China). Plasmid transfection was performed using Lipofectamine3000 (Invitrogen, USA) following the manufacturer’s protocol. GC cells (AGS, HGC-27, MKN45) were transfected with either control lentivirus (pLKO.1 or pCMV) or lentivirus containing LBP overexpression or knockdown constructs (Vigene Biosciences, Shandong, China) according to the manufacturer’s instructions. The GFP + HGC-27 cells were generated through transfection with a control lentivirus carrying GFP gene (Vigene Biosciences, Shandong, China). It is noteworthy that all lentiviral constructs used in this study were engineered to include the Firefly luciferase gene. Stable transfectants were selected using puromycin hydrochloride (MedChemExpress, Shanghai, China). The information regarding the shRNA utilized can be found in Supplementary Table 3.

### Western blot

The sample tissues and cultured cells were lysed with RIPA buffer (Beyotime, Shanghai, China) supplemented with protease inhibitor cocktails (HY-K0010, MedChemExpress, Shanghai, China) and phosphatase inhibitor cocktails (HY-K0023, MedChemExpress, Shanghai, China), then centrifugated at 15,000 g for 15 min at 4 °C. The protein concentration was measured using the BCA protein assay kit (Thermo Fisher, MA, USA). The proteins were denatured at 100 °C for 5 min in SDS-PADE protein buffer (Beyotime, Shanghai, China), and separated on 7.5–15% SDS–polyacrylamide gels. After the cells were incubated in serum-free medium for 24 h at 37 °C, the conditioned medium (CM) was collected by centrifugation at 2,000 g for 10 min at 4 °C. TCA (T0699, Sigma-Aldrich, USA) precipitation was performed to collect the secreted proteins in the CM, as previously described [24]. The antibodies used in WB are listed in Supplementary Table 4.

### Flow cytometry

The primary liver mixed cells were isolated as previously described [25]. Briefly, the livers were perfused with HBSS via the portal vein after the mice were euthanized. The livers were then collected, rinsed, minced, followed by digestion in DMEM supplemented with Collagenase Type IV (1 mg/ml, Sigma-Aldrich, USA), DNase (150 U/ml, Roche, Germany), and Dispase (1 U/ml, Invitrogen, USA) at 37 °C for 30 min. The cell suspension was filtered through 70-μm nylon strainers (Corning, USA) and washed with DMEM three times. Red blood cells were removed using RBC lysis reagent (Biosharp, China). To characterize myeloid cell subsets, appropriate antibodies were used to stain the cells after they were washed three times with PBS supplemented with 0.2 mM EDTA and 2% FBS. Flow cytometry analysis was performed using the CytoFLEX (Beckman, USA) system and quantified with the CytExpert software.

### ELISA

ELISA kits were used to determine the levels of human RBP4 (KE00056, Proteintech, China), human LBP (KE00134, Proteintech, China), human TGF-β1 (BMS249-4, Thermo Fisher Scientific, USA) in serum samples or cell culture supernatants, following the manufacturer’s instruction. Signal detection was performed using spectrophotometers (Thermo Fisher Scientific, USA) at 450 nm.

### Haematoxylin–eosin (H&E) staining and immunohistochemistry (IHC) staining

Standard IHC staining and H&E staining were performed on 4-μm formalin-fixed paraffin-embedded (FFPE) sections or 6-μm OCT-embedded cryosections, as previously described [26]. For IHC staining, tissues were incubated with primary antibodies overnight at 4 °C, subsequently, immunodetection was performed using DAB (DAKO) according to the manufacturer’s instructions. The Nikon Eclipse E600 Scanner system was used to capture images of the histology and IHC staining. The IHC scoring followed the methodology established in a prior study [27]. In brief, Negative staining was defined as the complete absence of staining or weak staining in less than 10% of the tumor cells. Weak staining indicated weak expression in more than 10% of the tumor cells. Medium staining represented moderate or strong expression in 10% to 50% of the tumor cells. High staining corresponded to moderate or strong expression in more than 50% of the tumor cells. The information regarding the antibodies utilized can be found in Supplementary Table 4.

### Immunofluorescence (IF) staining

For IF staining on FFPE sections, tissues were deparaffinized and subjected to antigen retrieval with retrieval solution (Beyotime Biotechnology, China) in a pressure cooker for 20 min. Deparaffinization and antigen retrieval were unnecessary for IF staining on cryosections, Tissues were blocked with 5% BSA in PBS containing 0.1% TWEEN 20 (PBS-T) for 30 min at room temperature (RT). For cultured cell IF staining, cells were fixed with 4% paraformaldehyde for 20 min, washed with PBS for three times, and permeabilized with or without 0.1% Triton X-100 in PBS for 15 min, followed by blocking in 2% BSA in PBS-T for 30 min at RT. Subsequently, the samples were incubated with primary antibodies overnight at 4 °C. Fluor-conjugated secondary antibodies were then applied to the samples for 2 h at RT. Filamentous actin (F-actin) was detected using Phalloidin-488 (A12379, Invitrogen) or Phalloidin-555 (A34055, Invitrogen). The nuclei were counterstained with DAPI. Fluorescence images were captured using by Stellaris STED laser scanning confocal microscopy system (LEICA, Germany). The information regarding the antibodies utilized can be found in Supplementary Table 4.

### Recombinant LBP production and purification

The pET28a expression vector (Novagen) was used to produce recombinant full-length LBP fused with GFP and His at the C terminus, as previously described [28]. Briefly, Escherichia coli BL21(DE3) was used to amplify the plasmid, which was then lysed in lysis buffer (20 mM sodium phosphate with 10 mM imidazole, 0.5 M NaCl and EDTA-free protease inhibitors, PH 7.5). The purification of the His-tagged proteins was carried out utilizing the His Ni–NTA Purification Kit (Thermo Scientific, 88,229), in accordance with the manufacturer's guidelines. Subsequently, endotoxins were removed using Pierce High-Capacity Endotoxin Removal Resin (Thermo Fisher, MA, USA), following the manufacturer's instructions.

### mRNA sequencing

mRNA sequencing was performed and analyzed on PMA-treated THP-1 cells with or without reLBP treatments using the platform of BGIseq 500 (BGI-Shenzhen, China). The mRNA sequencing data are presented in Supplementary Table 5.

### Immunoprecipitation and mass spectrometry

Immunoprecipitation of LBP fused with 3X-FLAG in THP-1 cells was performed using anti-FLAG M2 Affinity Gel (A2220, Sigma, USA) according to the manufacturer’s protocol. The eluted samples were then run on Bolt 10% Bis–Tris Plus Gel (Invitrogen) and stained using Fast Silver Stain Kit (Beyotime Biotechnology, Shanghai, China). Finally, mass spectrometry (OE Biotechnology, Shanghai, China) was used to determine the proteins that interacted with LBP. The mass spectrometry data are presented in Supplementary Table 6.

### 5-Ethynyl-2’deoxyuridine (EdU) assay

The proliferation of GC cells was assessed using the EdU assay kit (RiboBio, Guangzhou, China) according to the manufacturer’s protocol. Briefly, 1 × 10^4^ GC cells were seeded into 48-well plates and cultured for 24 h. The cells were then treated with 50 μM EdU for 2 h, fixed with 4% paraformaldehyde, and stained with Apollo Dye Solution and Hoechst 33,342. Fluorescence images were acquired using the THUNDER DMi8 fluorescence microscopy system (LEICA, Germany), with 10 random fields (200x) captured per well.

### Wound-healing assay

GC cells were cultured in 6-well plates. After 24 h of cell growth, a wound was gently scratched through the center of the cell monolayer using a pipette tip. The residual cell debris was carefully washed with serum-free medium for three times. Subsequently, the cells were cultured in the medium with l% FBS. Images were acquired at time points 0 h, 24 h, and 48 h using a microscope. The migration rate of cells in each experimental group was calculated based on the area covered by migrated cells.

### Transwell assay

The migration and invasion of GC cells were validated using transwell chambers (8 μm, Corning, USA) coated without or with matrigel (Corning, NY, USA), respectively. Briefly, 2 × 10^4^ GC cells in 200ul serum-free medium were seeded in the upper chamber, while 700 μl medium with 10% FBS was added to the lower chamber as the chemoattractant. After 48 h of incubation, the cells in the upper chamber were fixed with 4% formaldehyde and stained with 0.1% crystal violet. The migrated cells on the outer side of the upper chamber were counted after the remaining cells on the inner side of the upper chamber were removed. Six random fields (100x) were analyzed per well to determine the number of migrating cells.

### Animal experiments

BALB/c nude mice aged between 4 to 6 weeks, obtained from the Laboratory Animal Centre of Nanjing Medical University, were used for all animal experiments of this study. The mice were raised under pathogen-free conditions and all procedures were reviewed and approved by the Animal Ethics Committee of Nanjing Medical University (IACUC-2207061). The mice were divided into appropriate groups based on the experimental design, with age- and gender- matching taken into consideration.

To analyze the liver metastatic burden of GC cells, we developed a novel modified intrasplenic injection mouse model of liver metastasis. Briefly, mice were anesthetized with isoflurane and the abdomen was sterilized. A laparotomy (0.5–0.8 cm) was performed on the left upper abdomen to expose the spleen. The spleen was carefully exteriorized out of peritoneal cavity, and a surgical knot was pre-prepared at the end of spleen without ligating the pancreas. Firefly luciferase-labeled GC cells (1 × 10^6^ cells suspended in 100 μl PBS) were then injected into the spleen, away from the surgical knot, using a 30-gauge needle. After successful injection, the surgical knot was ligated the injection point was sterilized with 75% ethanol to prevent haemorrhage and leakage of tumor cells. The spleen was then gently placed back into the peritoneal cavity, and the peritoneum and skin were closed using a 3–0 absorbable suture (Ethicon) respectively. After surgery, buprenorphine (0.05–0.1 mg/kg) was subcutaneously administered to the mice every 6–8 h for 3 days. The progression of liver metastasis was monitored once a week using an in vivo imaging system (Calliper Life Sciences, Hopkinton, MA, USA). Bioluminescent detection was achieved through intraperitoneal injection of the substrate (D-Luciferin potassium salt, Gold Biotechnology, USA). The mice were sacrificed in 4th or 5th week after surgery, and their livers and spleens were collected for further analysis.

For the ‘pre-modelling’ model of LM, PBS, IgG (100 μg per mouse) or reLBP (100 μg per mouse) in 100 μl PBS was injected into mice via the tail vein every two days for two weeks. The livers were collected to evaluate intrahepatic PMN formation. Additionally, mice were intrasplenically injected with firefly luciferase-labeled GC cells to evaluate the extent of liver metastatic burden following pre-modelling.

To deplete intrahepatic F4/80^+^ myeloid cells while receiving reLBP pre-modelling, mice were administered clodronate-encapsulated liposomes (CEL, Liposoma) via intraperitoneal injection using a 30-gauge needle, according to the manufacturer’s instructions. Mice receiving reLBP pre-modelling were also administered anti-LBP antibodies (R&D-Biotechne) at a dose of 200 μg suspended in 100 μl of PBS via intraperitoneal injection using a 30-gauge needle every two days until the point of sacrifice. Galunisertib (also known as LY2157299, MedChemExpress, Shanghai, China) was also administered to mice by oral gavage at a dose of 75 mg/kg twice daily after receiving reLBP pre-modelling until the point of sacrifice.

For the subcutaneous xenograft model, GC cells with LBP stable knockdown or overexpression (1 × 10^6^ cells per mouse) were subcutaneously injected into the axilla of the forelimb. The tumor volumes (V = length × width^2^ × 0.5) were measured once a week for 4–5 weeks. The subcutaneous tumors were collected for further analysis after the mice were sacrificed at the final time point.

### Statistical analysis

As stated in the figure legends, all in vitro experiments were independently performed more than three times with consistent results, and the data were presented as mean ± SD or mean ± SEM. GraphPad Prism 9.0 (GraphPad Software, La Jolla, USA) was utilized for data analysis. Differences between two groups were determined by performing a two-tailed unpaired student's t-test. For comparisons among multiple groups, a one-way or two-way ANOVA test was conducted. Survival analysis was performed using the Log-rank test or Kaplan–Meier method. The *p*-value < 0.05 was considered statistically significant.

## Results

### LBP secretion is associated with GC metastasis to the liver and correlated with a worse prognosis in patients with GC

**Fig. 1:**
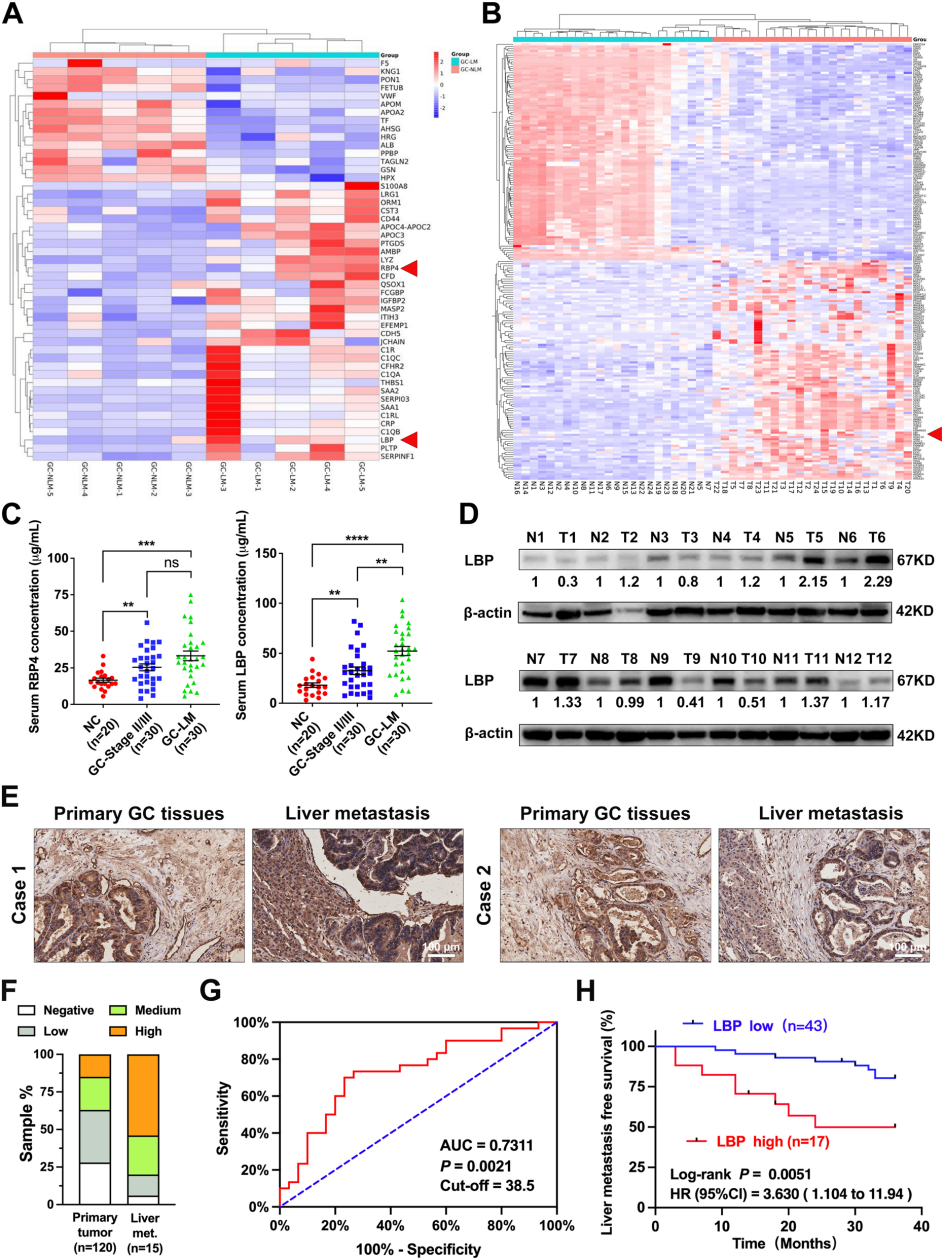
LBP secretion is associated with GC metastasis to liver and correlated with worse prognosis. **A** Heatmap of 49 dysregulated secreted proteins detected by DIA-MS secretomic analysis of serum samples from five patients with GC-LM and five patients with GC-NLM. **B** Clustered heatmap analysis of the top 200 dysregulated mRNAs of 24 pairs of GC tissues and matched normal gastric tissues (|foldchange|> 2; *p* < 0.05). **C** Serological levels of LBP and RBP4 were detected by ELISA in the serum of 30 patients with GC-LM, 30 patients with stage II/III GC and 20 healthy volunteers. **D** WB verified the expression levels of LBP in GC tissues and matched adjacent normal gastric tissues. **E** Representative IHC images of LBP in paired primary tumours and LM from the same GC patients. Scale bar, 100 μm. **F** Comparisons of LBP expression in primary GC tumors and LM based on IHC results. **G** ROC curve analysis was applied based on the previous serological LBP levels of 30 GC-LM patients and 30 GC-NLM patients. **H** Liver metastasis-free survival analysis of an additional cohort of GC patients based on serological LBP levels. The cut-off value was determined according to the ROC curve. Data are representative of three independent experiments. Data are shown as mean ± SEM, and *p* values were determined by one-way ANOVA test (**C**) or log rank test (**H**) (* *P* < 0.05, ** *P* < 0.01, *** *P* < 0.001)

To identify potential tumour-secreted proteins associated with GC-LM, DIA-MS was performed on the serum samples from five patients with GC-LM and five patients with GC-NLM. Overall, 49 dysregulated secreted proteins were detected in both groups, as illustrated in Fig. 1A. Additionally, mRNA expression microarrays analysis of 24 pairs of GC and matched normal gastric mucosa was conducted in our previous study to identify the most dysregulated genes in GC (Fig. 1B) [22]. Through an overlapping analysis (Supplementary Fig. 1A), two promising secreted proteins (LBP and RBP4) were identified. Subsequently, LBP was validated as the most significantly secreted protein associated with GC-LM by using ELISA (Fig. 1C) and qRT-PCR (Supplementary Fig. 1B). WB and IHC were performed on a cohort of patients with GC, and it was found that the GC tissues showed higher LBP expression than the adjacent normal tissues (Fig. 1D and E; Supplementary Fig. 1C and D). Moreover, it was observed that LBP was upregulated in primary GC-LM tissues compared to GC-NLM tissues (Supplementary Fig. 1C), and LM tissues had even higher LBP expression than primary tumours (Fig. 1E and F). Furthermore, data from TCGA dataset showed that LBP expression was correlated with a poor prognosis in terms of the disease-free survival (Supplementary Fig. 1E) and overall survival (Supplementary Fig. 1F) of patients with GC. ROC curve analysis of previous serological LBP levels of 30 patients with GC-LM and 30 patients with GC-NLM indicated that serological LBP level could potentially be used as a diagnostic biomarker of GC-LM (Fig. 1G, AUC = 0.7311, *p* = 0.0021). LM-free survival analysis was further performed in an additional cohort of patients with GC, revealing that patients with higher serological LBP levels were more likely to develop liver metastasis after curative gastrectomy (Fig. 1H). Collectively, these results demonstrate that GC-derived LBP is associated with GC-LM and is correlated with a worse prognosis in patients with GC.

### LBP promotes GC-LM

**Fig. 2:**
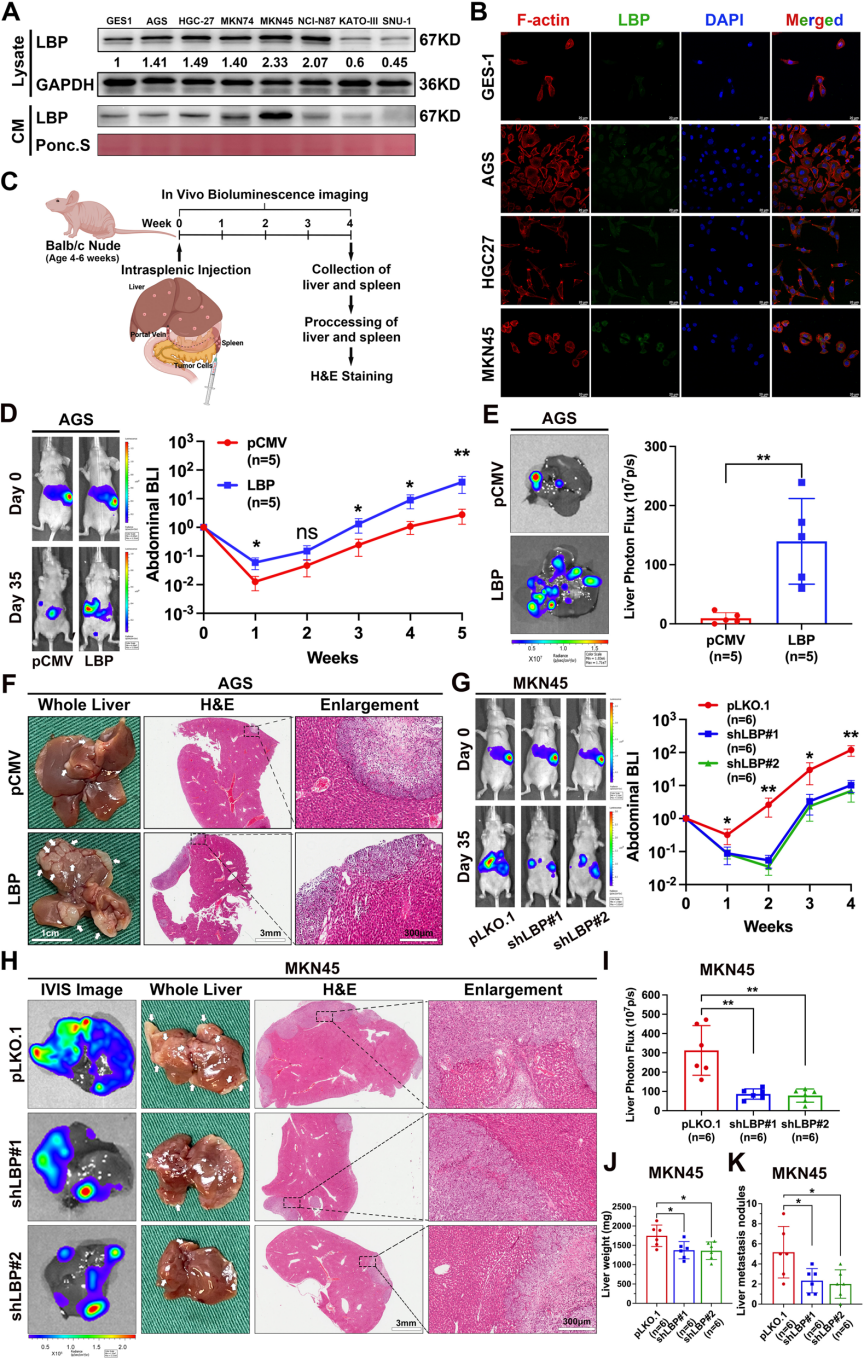
LBP promotes GC-LM in vivo. **A** Protein level of LBP was determined in the cell lysate and CM of GC cell lines and normal gastric epithelial cell line (GES-1) by WB. Ponc. S staining was used as the loading control of CM. CM, conditioned medium. Ponc. S, Ponceau S staining. **B** Representative IF images of LBP expression in GES-1 and GC cells (AGS, HGC-27, MKN45). **C** Schematic diagram of intrasplenic injection mouse model of LM and experimental workflow. The image was drawn by using BioRender.com. **D** Representative bioluminescent images (left) and BLI quantification (right) of mice with intrasplenic injection of AGS cells (1 × 10^6^ cells per mouse) with or without LBP stable overexpression for LM analysis (*n* = 5 mice per group for control and overexpression). **E** Mice were euthanized in the 5th week after intrasplenic injection. Representative bioluminescent images (left) and photon flux quantification (right) of LM are shown.** F** Representative photographs (left) and H&E staining with enlargement (middle, right) of LM are shown. **G-K** Intrasplenic injection of MKN45 cells (1 × 10.^6^ cells per mouse) with or without LBP stable knockdown for LM analysis (*n* = 6 mice per group). Representative bioluminescent images (**G**, left) and BLI analysis (**G**, right) of mice are shown. Mice were euthanized in the 4th week after intrasplenic injection. Representative bioluminescent images (**H**, column of IVIS image), photographs (**H**, column of whole liver) and H&E staining with enlargement (**H**, columns of H&E and enlargement) of LM are shown, respectively. Quantification of liver photon flux (**I**), liver weight (**J**) and surface metastatic nodules of liver (**K**) was applied. Scale bars are 20 μm (**B**), 1.0 cm (**F** left, **H** column 2), 3 mm (**F** middle, **H** column 3), 300 μm (**F** right, **H** column 4), and the colored scale bars represent the intensity of luminescence as indicated, respectively. Data are representative of three independent experiments. Data are shown as mean ± SEM (**D**, **G**), or mean ± SD (**E**, **I**,** J**, **K**). *p* values were determined by two-way ANOVA test (**D**, **G**), two-tailed unpaired Student’s t test (**E**), or one-way ANOVA test (**I**,** J**, **K**) (*P < 0.05, **P < 0.01, ***P < 0.001)

To determine the expression levels of LBP in GC cell lines, WB and qRT-PCR were performed on seven GC cell lines and a normal gastric epithelial cell line (GES-1). The results indicated that most gastric cell lines had higher LBP expression levels compared to those of GES-1 cells (Fig. 2A and Supplementary Fig. 2A). Furthermore, the conditional medium (CM) from MKN45 cells contained the highest level of LBP (Fig. 2A). These results were confirmed by IF staining (Fig. 2B). Next, AGS (a GC cell line with a low level of endogenous LBP) was constructed with stable LBP overexpression, and MKN45 (a GC cell line with a high level of endogenous LBP) was constructed with stable LBP knockdown using short hairpin RNAs, respectively, which were verified by qRT-PCR (Supplementary Fig. 2B and C) and WB (Supplementary Fig. 2D). A novel modified intrasplenic injection mouse model of LM (Supplementary Fig. 2E) was developed to validate the functional role of LBP in LM in vivo in BABL/c nude mice (Fig. 2C). Notably, in vivo bioluminescence imaging (BLI) performed once a week, revealed that LBP overexpression in AGS cells significantly exacerbated the progression of LM after intrasplenic injection (Fig. 2D). Mice were euthanised in the 5th week after intrasplenic injection, and the livers and spleens were collected for the analyses of photon flux quantification, weighing, H&E staining, and counting of metastatic nodules on the liver surface, respectively. Consistently, LBP overexpression in AGS cells significantly enhanced liver-metastatic burden (Fig. 2E and F; Supplementary Fig. 2F and G). In contrast, LBP knockdown by short hairpin RNAs in MKN45 cells significantly inhibited the progression of LM (Fig. 2G) and reduced liver-metastatic burden (Fig. 2H-K). These results demonstrated the pro-metastatic role of LBP in GC-LM.

### LBP promotes GC cell colonisation and outgrowth in the liver at early stages by inducing the intrahepatic fibrotic PMN

**Fig. 3:**
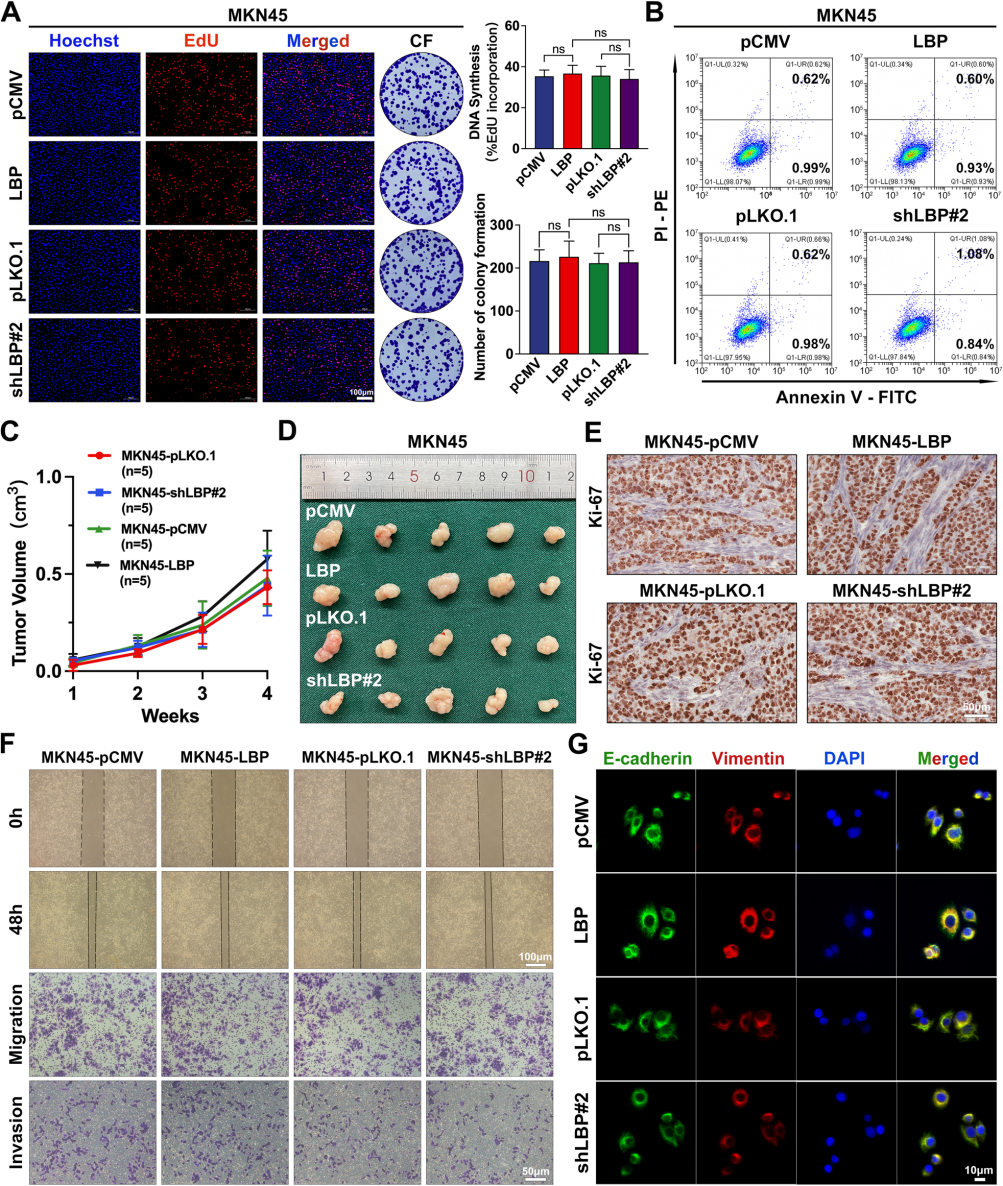
Endogenous LBP has no significant effects on proliferation, apoptosis, invasion and migration, and mesenchymal transition of GC cells. **A** The effect of LBP on the proliferation in MKN45 cells was explored by EdU and colony formation assay with LBP stable overexpression or knockdown. Data are shown as mean ± standard deviation of 3 biologically independent experiments, however, the *p* values are not statistically significant. **B** Flow cytometry analysis of apoptosis was determined in MKN45 cells with stable LBP overexpression or knockdown. **C** The volumes of tumours were measured once a week after subcutaneous injection with MKN45 cells with stable LBP overexpression or knockdown. The growth curves of tumours are shown as mean ± SEM at sequential timepoints (*n* = 5 per group). **D** Photograph of subcutaneous tumours after mice were sacrificed in the 4th week. **E** Representative IHC images of Ki67 in subcutaneous tumours with LBP stable overexpression or knockdown. **F** Wound-healing assays (row 1, 2) and Transwell assays (row 3, 4) were applied to validate the effect of LBP on the migration and invasion of MKN45 cells. **G** Representative immunofluorescence images of EMT markers in MKN45 with LBP stable overexpression or knockdown. E-cadherin (red), Vimentin (green), and DAPI (blue). Scale bars are 100 μm (**A**,** F**), 50 μm (**E**), or 10 μm (**G**). Data are representative of three independent experiments. *p* values were determined by one-way ANOVA test (**A**) or two-way ANOVA test (**C**) (ns, not significant)

To investigate the mechanism through which LBP promotes GC-LM, its effect on the intrinsic malignancy of tumour cells was first examined. EdU and colony formation assays were performed to assess the effects of LBP on the proliferation of MKN45 and HGC-27 cells (a GC line with a moderate level of endogenous LBP) following LBP overexpression or knockdown. These results indicated that LBP had no significant effect on the proliferation of GC cells (Fig. 3A and Supplementary Fig. 3A). Similarly, LBP had no significant effect on apoptosis of GC cells, as determined by the flow cytometry analysis of MKN45 and HGC-27 cells (Fig. 3B and Supplementary Fig. 3B), and tumour sphere formation was also unaffected by LBP overexpression or knockdown (Supplementary Fig. 3C). Consistently, subcutaneous inoculation of xenograft tumours further confirmed that LBP had no significant effect on the growth of subcutaneous tumours in vivo (Fig. 3C and D), and IHC staining of Ki-67 showed that LBP had no significant effect on the proliferation of subcutaneous tumours (Fig. 3E). In addition, wound-healing, Transwell assays, IF, and WB indicated that LBP had a slight impact on the invasion, migration, and epithelial-mesenchymal transition (EMT) of GC cells (Fig. 3F-G; Supplementary Fig. 3D-J). Therefore, it was proposed that LBP promotes GC-LM in a microenvironment-dependent manner.

**Fig. 4:**
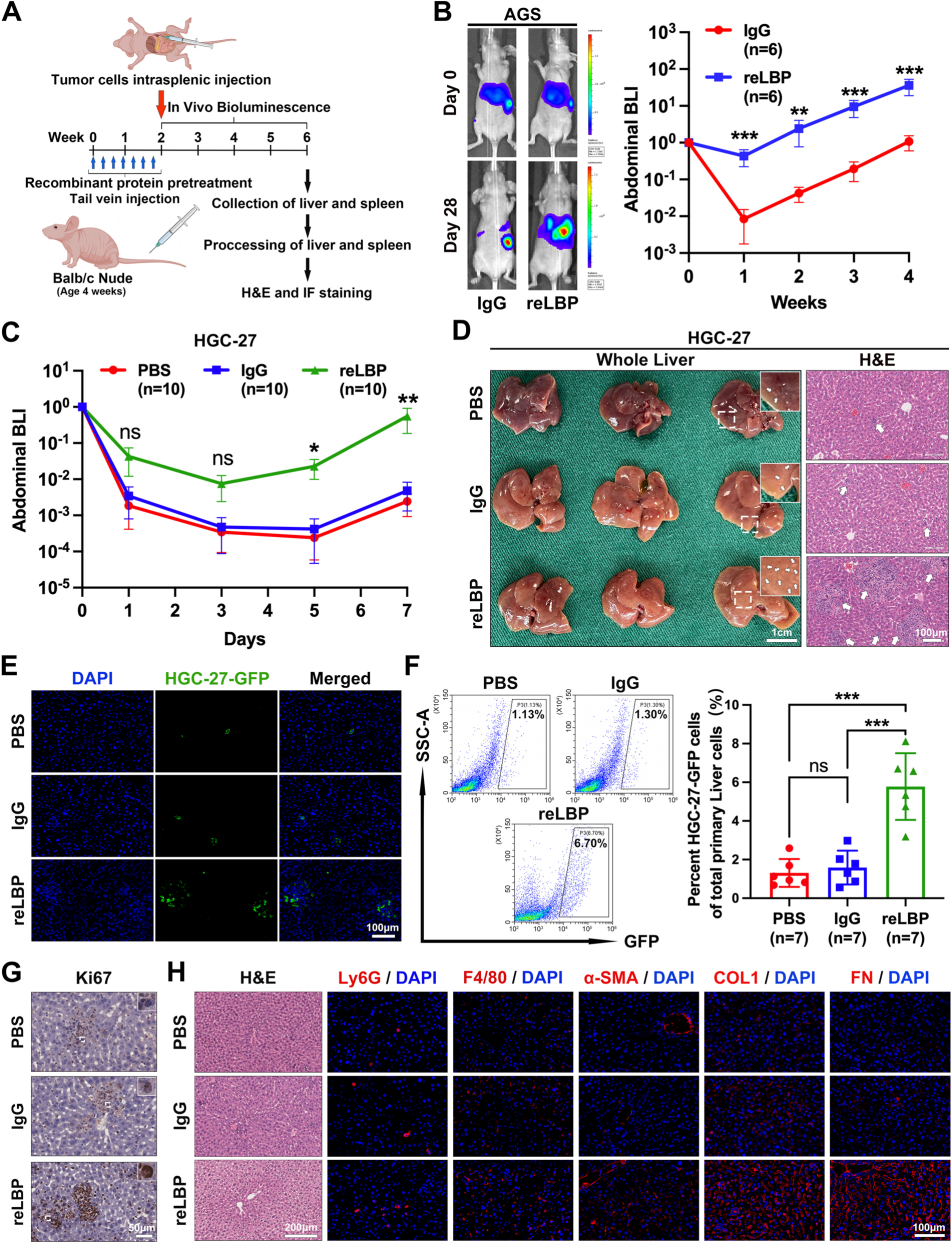
LBP promotes GC cell colonization and outgrowth in the liver at the early stages by inducing intrahepatic fibrotic PMN formation in pre-modelling mouse model. **A** Schematic diagram of pre-modelling mouse model of LM and experimental workflow. The image was drawn by using BioRender.com. **B** Representative bioluminescent images (left) and BLI analysis (right) of mice with intrasplenic injection of AGS cells (1 × 10^6^ cells per mouse) after mice were pretreated with reLBP or IgG for two weeks (*n* = 5 mice per group). **C-G** Colonization and proliferation of cancer cells in the liver at the early stage (in the first week) with intrasplenic injection of HGC-27 cells after mice were pretreated with PBS, IgG or reLBP, respectively (*n* = 10 mice per group). BLI quantification (**C**) are shown by comparing the bioluminescence at sequential timepoints. Mice were euthanized on the 7th day after intrasplenic injection. Representative photograph and H&E staining (**D**), IF analyses of GFP^+^ HGC-27 cells in the liver sections (**E**), flow cytometry analysis of GFP.^+^ HGC-27 cells in the liver (**F**), and representative IHC images of Ki67 in tumour cells in the liver are shown. **H** Representative H&E and IF images of myeloid cells (Ly6G, F4/80), α-SMA, FN, and COL1 in the livers after mice were pretreated with PBS, IgG or reLBP for two weeks, respectively. Scale bars are 1.0 cm (**D**, left), 200 μm (**H**, H&E), 100 μm (**D**-right, **E**, **H**-IF) and 50 μm (**G**), the colored scale bars represent the intensity of luminescence as indicated, respectively. Data are representative of three independent experiments. The BLI quantification is shown as mean ± SEM at sequential timepoints (**B**, **C**). Data of flow cytometry analysis are shown as mean ± SD (**F**). *p* values were determined by two-way ANOVA test (**B, C**) or one-way ANOVA test (**F**) (ns, not significant, * *P* < 0.05, ** *P* < 0.01, *** *P* < 0.001)

To confirm this hypothesis, a mouse model was developed using pre-modelling with recombinant proteins to demonstrate the effect of exogenous LBP on LM and the intrahepatic microenvironment (Fig. 4A). After mice were pretreated with recombinant LBP (reLBP) or IgG by tail vein injection once every other day for 2 weeks, mice were intrasplenically injected with luciferase-labelled GC cells. As shown in Fig. 4B, exogenous LBP significantly exacerbated the progression of LM after intrasplenic injection of AGS cells, ultimately leading to a significant increase in the liver-metastatic burden (Supplementary Fig. 4A-C). Moreover, consistent results were observed after intrasplenic injection of MKN45 cells (Supplementary Fig. 4D and E). Notably, BLI analyses showed that pre-modeling with exogenous LBP significantly increased cancer cell colonisation in the liver, even in the first week after intrasplenic injection (Fig. 4B and Supplementary Fig. 4D). Furthermore, daily analysis of bioluminescence signals within the first week after the intrasplenic injection of HGC-27 cells consistently indicated that LBP increased the colonisation and outgrowth of GC cells in the liver at an early stage (Fig. 4C). Mice were euthanised on the 7th day after intrasplenic injection. Representative photographs and H&E staining revealed increased GC cell seeding and outgrowth in the liver with reLBP pre-modelling (Fig. 4D). This result was further confirmed by IF staining and flow cytometric analysis of the liver (Fig. 4E-F). In addition, Ki-67 IHC staining of liver sections suggested that reLBP pre-modelling induced the proliferation of GC cells at the seeding stage in the liver (Fig. 4G). Therefore, the results suggested that exogenous LBP induces a PMN to facilitate GC cell seeding and outgrowth in the liver in the early stages.

As previously reported [29, 30], myeloid cell accumulation and ECM deposition are identified as key features of the PMN. Therefore, the changes in intrahepatic stromal infiltrated myeloid cells and ECM were examined after the mice were treated with PBS, IgG and reLBP by tail vein injection once every other day for two weeks, respectively. As expected, the livers of mice pre-modelling with reLBP presented increased activation of HSCs (α-smooth muscle actin, α-SMA) accompanied by significant increased deposition of type I collagen (COL1) and fibronectin (FN) as compared to that of control mice, which was determined by IF and IHC staining (Fig. 4H and Supplementary Fig. 4I). Interestingly, reLBP induced angiogenesis in the liver (Supplementary Fig. 4J). Although it was observed that the livers of reLBP-treated mice contained more infiltrated myeloid cells than control mice, the difference was not significant (Fig. 4H), as confirmed by fluorescence-activated cell sorting (FACS) analyses (Supplementary Fig. 4F-H). Our data indicated that myeloid cells are not essential for ECM deposition in the liver, which is consistent with the findings of previous studies [14, 25]. WB analysis further confirmed that reLBP pre-modelling significantly promoted ECM deposition in the liver, as demonstrated by the upregulation of FN, COL1, and α-SMA, and the downregulation of vitronectin and tenascin C (Supplementary Fig. 4K). As well known, HSCs mainly mediate intrahepatic ECM remodeling. IF staining of primary HSCs isolated from the livers of mice treated with PBS, IgG, and reLBP was further performed. It was found that the HSCs from reLBP-treated mice were markedly activated (Supplementary Fig. 4L). Collectively, our results demonstrated that LBP promotes GC cell seeding and outgrowth in the liver at the early stages by inducing intrahepatic fibrotic PMN formation.

### Intrahepatic macrophages mediate the PMN formation induced by LBP

**Fig. 5:**
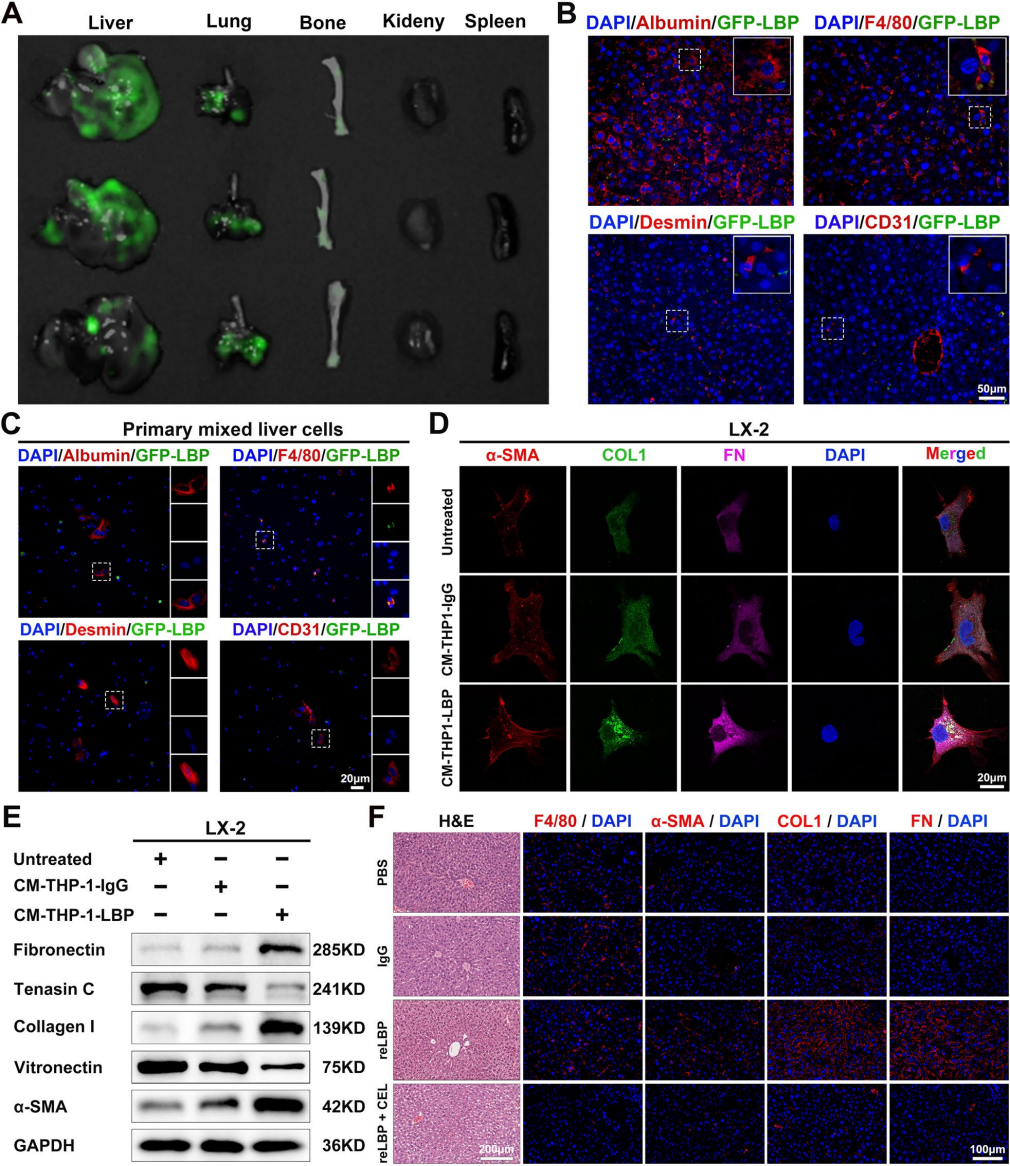
GC-derived LBP are mainly accumulated in the liver and colocalized with intrahepatic macrophages. **A** Representative bioluminescent images of the liver, lung, bone, kidney and spleen tissues at the 24th hour after tail injection of reLBP-GFP (200 μg). **B** Representative IF images with partial amplification are shown that reLBP-GFP were mainly colocalized with macrophages (F4/80^+^ cells) in mouse liver after reLBP-GFP tail vein injection. **C** reLBP-GFP were mainly colocalized with macrophages (F4/80^+^ cells) in primary mixed liver cells in vitro. **D** Representative IF images shown that the CM from reLBP-pretreated THP-1 cells indues overexpression of fibrotic markers in HSC cell line (LX-2). **E** WB was applied to confirm that the CM from reLBP-pretreated THP-1 cells activated LX-2. **F** Representative H&E and IF images of F4/80, α-SMA, FN, and COL1 in the liver after pre-modelling with PBS, IgG, reLBP, reLBP combined with macrophage depletion, respectively. Data are representative of three independent experiments. Scale bars are 50 μm (**B**), 20 μm (**C, D**), 200 μm (**F**–H&E) and 100 μm (**F**-IF). Data are representative of three independent experiments

To elucidate the mechanisms underlying LBP-induced PMN formation, GFP-labelled reLBP (GFP-LBP) was first injected into the mice via the tail vein to investigate the organotropism of LBP (Supplementary Fig. 5A). Consistent with expectations, analysis of fluorescence intensity analysis revealed that the liver had a significantly higher accumulation of GFP-LBP than other organs (Fig. 5A and Supplementary Fig. 5B), which was further confirmed by the IF staining of mouse organ tissues (Supplementary Fig. 5B). Furthermore, the main cell types in the liver were characterized by IF staining to identify the ‘recipient’ cells of LBP. Interestingly, it was found that LBP was mainly co-localized with F4/80^+^ Kupffer cells (KCs) (Fig. 5B), but not with HSCs (desmin-positive cells). Moreover, primary mixed liver cells isolated from untreated mice were incubated with GFP-LBP proteins in vitro, and the results indicated that GFP-LBP was mainly ‘taken up’ by macrophages (F4/80^+^ cells) (Fig. 5C). Subsequently, THP-1 cells (a human leukemia monocytic cell line) were induced into macrophages using phorbol-12-myristate-13-acetate (PMA) to study intrahepatic macrophages in vitro. As shown in Supplementary Fig. 5D and 5E the induced M0-like macrophages presented an adherent spread morphology with increased granularity and CD68 expression. Moreover, IF staining showed that GFP-LBP was ‘taken up’ by the PMA-treated THP-1 cells in vitro (Supplementary Fig. 5G). These results suggested that intrahepatic macrophages may play an essential role in PMN induced by LBP. To confirm this speculation, LX-2 (a human HSC line) cells were treated with the CM from reLBP-treated THP-1 cells in vitro. IF staining showed that the CM of reLBP-treated THP-1 significantly increased the expression of α-SMA, COL1, and FN in LX-2 cells compared to the control medium (Fig. 5D). These results were further confirmed by WB (Fig. 5E). Additionally, F4/80^+^ cells were depleted in the liver with clodronate-encapsulated liposomes (CEL) by intraperitoneal injection in the pre-modelling mouse model to determine intrahepatic ECM remodeling (Supplementary Fig. 5F). As expected, upon intrahepatic macrophage depletion, reLBP failed to induce the activation of HSCs and deposition of COL1 and FN in the liver (Fig. 5F). Collectively, these results demonstrate that intrahepatic macrophages mediate PMN induced by LBP.

### LBP activates the TLR4/NF-κB pathway in intrahepatic macrophages to promote TGF-β1 secretion, in turn, TGF-β1 activates HSCs to direct intrahepatic fibrotic PMN

**Fig. 6:**
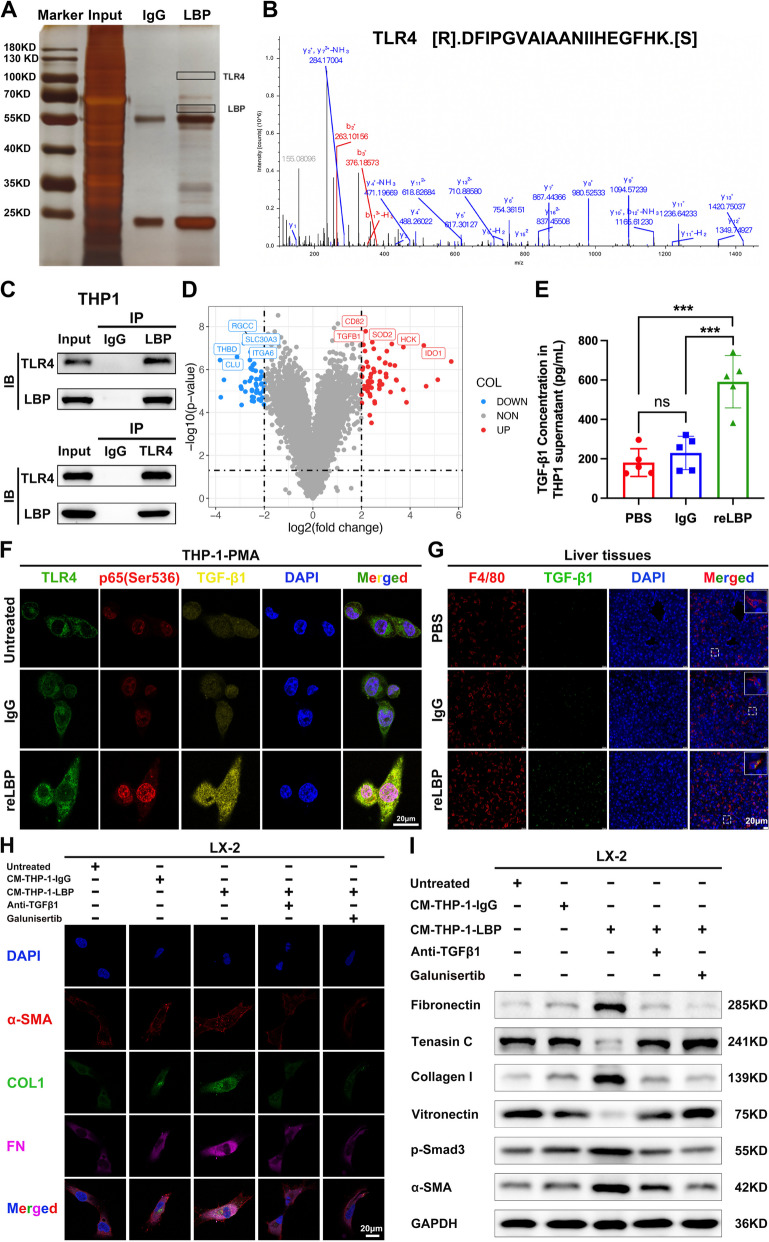
LBP activates the TLR4/NF-κB pathway in intrahepatic macrophages to promote TGF-β1 secretion, in turn, TGF-β1 activates HSCs to direct intrahepatic fibrotic PMN. **A** Representative image of silver staining showed the proteins that were pulled down by immunoprecipitation of LBP overexpression in THP-1 cells. **B** Representative secondary mass spectrum of TLR4 protein. **C** Representative IB images of CO-IP with LBP or TLR4 overexpression in THP1cells. **D** Volcano plot of mRNA sequencing in PMA-treated THP-1 cells with IgG vs reLBP pretreatment in vitro. **E** ELISA was applied to determine TGF-β1 concentration in the supernatant of PMA-treated THP-1 cells with PBS, IgG or reLBP education, respectively. **F** IF staining was performed to investigate the regulatory mechanism of LBP in THP1 in vitro. **G** Representative IF images of TGF-β1 secretion in macrophages in the livers of mice with PBS, IgG or reLBP education, respectively. **H-I** IF staining (**H**) and WB (**I**) were performed to conform that the CM from reLBP-pretreated THP-1 cells activated LX-2 to increase the markers of fibrosis, whereas the anti-TGF-β1 antibody and galunisertib inhibited TGF-β/Smad signaling pathway to block the activation of LX-2 induced by the CM from reLBP-treated THP-1 cells. Data are representative of three independent experiments. Data pooled as mean ± SD of 5 independent experiments (**C**), and *p* values were determined by one-way ANOVA test (* *P* < 0.05, ** *P* < 0.01, *** *P* < 0.001)

To further investigate the role of intrahepatic macrophages in PMN formation induced by LBP, immunoprecipitation assays were performed, followed by SDS-PAGE and mass spectrometry in PMA-THP-1 cells (Fig. 6A). Using an overlapping analysis of mass spectrometry results and predictions from the STRING databases (https://string-db.org/) (Supplementary Fig. 6A and 6B), TLR4 was identified as a promising receptor protein that interacts with LBP (Fig. 6B). Co-IP assays demonstrated an interaction between LBP and TLR4 after simultaneous overexpression of LBP and TLR4 in PMA-THP-1 cells (Fig. 6C). As shown in Supplementary Fig. 6C, protein–protein docking analysis also supported the interaction of LBP and TLR4 with high docking scores in the HDOCK SERVER database (http://hdock.phys.hust.edu.cn/). Additionally, the data from the Human Protein Atlas Database showed that KCs have a high expression of TLR4 (Supplementary Fig. 6D), which could partially explain the previous results indicating that the liver had greater LBP accumulation, and LBP was mainly ‘taken up’ by intrahepatic macrophages.

To identify the functional targets of LBP in macrophages, mRNA sequencing was performed to compare the gene expression profiles of PMA-THP-1 cells between IgG- and reLBP-treated groups. KEGG pathway analyses revealed that the NF-κB signaling pathway was one of the top-ranked pathways affected by reLBP (Supplementary Fig. 6E). Moreover, it was observed that TGF-β1 was among the top upregulated genes (Fig. 6D). As a well-known profibrotic cytokine, TGF-β1 is identified as an effective inducer of liver fibrosis [31, 32]. It was further confirmed that the supernatant from reLBP-treated THP-1 cells contained much more TGF-β1 protein than the control supernatant based on the ELISA (Fig. 6E). IF staining (Fig. 6F) and WB (Supplementary Fig. 6F) demonstrated that LBP upregulated the expression of TGF-β1 in PMA-THP-1 cells by activating the TLR4/NF-κB pathway. Concordantly, it was found that the livers of mice with reLBP pre-modelling presented significantly increased expression of TGF-β1 in intrahepatic macrophages (F4/80^+^cells) compared to those of control mice in vivo (Fig. 6G). Subsequently, anti-TGF-β neutralizing antibody and galunisertib (a selective TGF-β receptor type I kinase inhibitor) were applied to confirm whether TGF-β1 direct the activation of LX-2 cells in vitro. As expected, IF staining showed that anti-TGF-β neutralizing antibody and galunisertib attenuated the overexpression of α-SMA, COL1, and FN in LX-2 induced by the CM from reLBP-treated THP-1cells (Fig. 6I). Similarly, WB also demonstrated that anti-TGF-β1 antibody and galunisertib inhibited the TGF-β/Smad signaling pathway to block the activation of LX-2 induced by the CM of reLBP-treated THP-1 cells (Fig. 6J). Collectively, these results demonstrated that LBP activates the TLR4/NF-κB pathway in intrahepatic macrophages to promote TGF-β1 secretion, which, in turn, activates HSCs to direct fibrotic PMN formation in the liver.

### Selectively targeting the TGF-β/Smad pathway with galunisertib effectively prevents GC-LM in mice

**Fig. 7:**
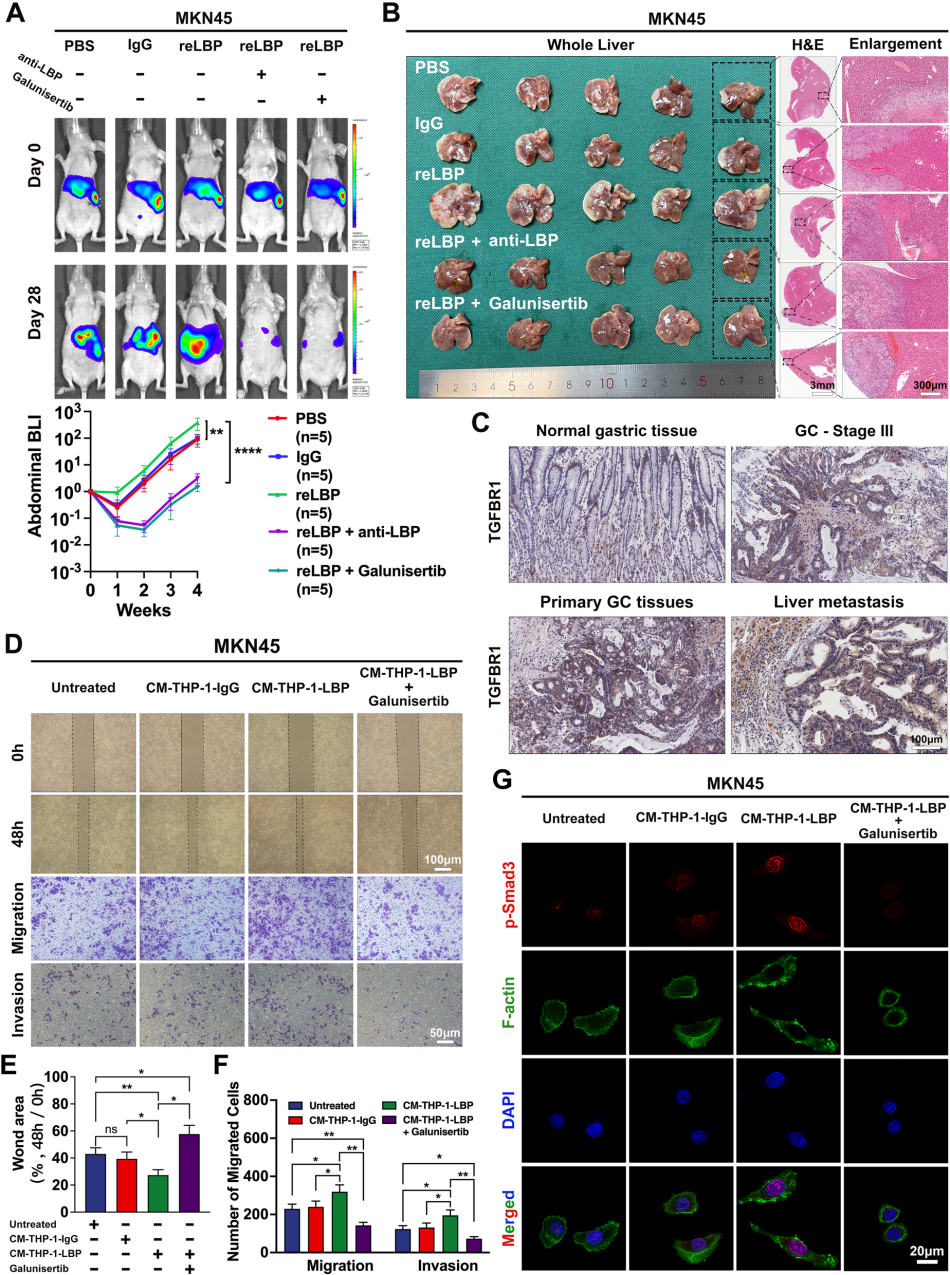
Selectively targeting TGF-β/Smad signaling pathway with galunisertib effectively prevents GC-LM in mice. **A** Representative bioluminescent images and BLI analysis of mice with intrasplenic injection of MKN45 cells (1 × 10.^6^ cells per mouse) showed the inhibitory effect of the anti-LBP antibody and galunisertib on LM in vivo (*n* = 5 per group). **B** Mice were euthanized in the 4th week after intrasplenic injection of MKN45 cells. Photograph and representative H&E with enlargement of LM are shown. **C**. Representative IHC images of TGFBR1 in human normal gastric tissue, stage III GC tissues, primary GC tissue and paired LM sections. **D-F** Wound-healing assays (row 1, 2) and Transwell assays (row 3, 4) were applied to validate the effect of CM from reLBP-treated THP-1 and galunisertib on the migration and invasion of MKN45 cells. **G** IF was applied to confirm that galunisertib inhibit the actin remodeling of MKN45 cells. Scale bars are 3 mm (**B**-middle), 300 μm (**B**-right) and 100 μm (**C**, **D-**wound-healing), 50 μm (**D-**Transwell assays), 20 μm (**G**). Data are representative of three independent experiments. Data are shown as mean ± SEM (**A**) or mean ± SD (**E**, **F**). *p* values were determined by two-way ANOVA test (**A**) or one-way ANOVA test (**E**, **F**) (ns, not significant, * *P* < 0.05, ** *P* < 0.01, *** *P* < 0.001)

Given the pivotal role of the TGF-β/Smad pathway in liver fibrosis induced by LBP, this study investigated whether targeting LBP or the TGF-β/Smad pathway could effectively prevent and treat GC-LM. The anti-LBP antibody and galunisertib, a selective small molecule inhibitor of TGF-β receptor I kinase, were tested in vivo. As expected, the anti-LBP antibody and galunisertib suppressed LBP-induced formation of the intrahepatic fibrotic PMN (Supplementary Fig. 7A). Moreover, both treatments significantly inhibited the progression of LM in mice with intrasplenic injection of MKN45 cells (Fig. 7A), eventually attenuating the liver-metastatic burden (Fig. 7B). Interestingly, galunisertib appeared to be more effective than the anti-LBP antibody in preventing GC-LM (Supplementary Fig. 7B), suggesting that galunisertib could inhibit GC-LM through an additional mechanism. The data from TCGA dataset showed that GC exhibited higher expression of TGFBR1 than normal gastric tissues (Supplementary Fig. 7C), and the expression levels of TGFBR1 were correlated with the progression of GC and a worse prognosis in patients with GC (Supplementary Fig. 7D and G). The Human Protein Atlas database also revealed high TGFBR1 expression in GC (Supplementary Fig. 7E). Consistently, IHC staining showed that liver metastatic tissues had a higher expression of TGFBR1 (Fig. 7C), whereas IF staining demonstrated that TGFBR1 was highly expressed in GC cell lines compared to GES-1 (Supplementary Fig. 7F). Furthermore, wound-healing and Transwell assays revealed that the migration and invasion of MKN45 cells were significantly enhanced by the CM of reLBP-treated THP-1 cells. However, galunisertib treatment markedly attenuated these effects (Fig. 7D-F). Similarly, it was found that the CM from reLBP-treated THP-1 cells significantly increased actin remodeling in MKN45 cells by activating the TGF-β/Smad signaling pathway, whereas galunisertib markedly inhibited this actin remodeling by downregulating the expression of p-Smad3 (Fig. 7G and Supplementary Fig. 7H). These results suggested that increased intrahepatic TGF-β1 induced by GC-derived LBP could enhance the migration and invasion of metastatic GC cells, further promoting the colonization of cancer cells in the liver. Collectively, the results demonstrated that selectively targeting the TGF-β/Smad signaling pathway with galunisertib effectively prevents GC-LM by inhibiting fibrotic PMN formation and attenuating the migration and invasion of metastatic GC cells in the liver.

## Discussion

LM, one of the most common haematological spread patterns of GC, stands as a primary contributor to GC-related mortality [33]. Moreover, a poor understanding of the underlying mechanisms of GC-LM greatly contributes to its high mortality rate. In this study, LBP was identified as a novel and critical secreted protein associated with GC-LM. Mechanistically, GC-derived LBP drives intrahepatic fibrotic PMN formation by inducing TGF-β1 secretion in intrahepatic macrophages to promote GC-LM.

Diagnosis of GC-LM currently relies on enhanced CT, MRI, PET-CT, and biopsy pathological diagnosis, which can only detect ‘visible’ metastatic lesions [34]. However, primary tumours can initiate metastasis before spreading from primary sites by inducing PMN formation via soluble factors [35]. Recently, PMNs have attracted considerable attention owing to their critical roles in determining metastatic efficacy [36, 37]. Small extracellular vesicles originating from cancer cells have been extensively recognized as initiators of PMNs by delivering proteins, RNA, and DNA in various cancer types [38, 39]. Consistently, our previous findings indicated that GC-derived extracellular vesicles can initiate a hepatic stemness-enhancing niche by delivering miR-151a-3p, thereby facilitating the promotion of GC-LM [40]. Compared with small extracellular vesicles, secreted proteins containing a signal peptide are more accessible and cost-effective for detection in serum. They are also recognized as pivotal initiators capable of inducing PMNs. Bastian Seubert et al. demonstrated that pancreatic precancerous lesions secrete tissue inhibitor of metalloproteinase-1 (TIMP1), which activates HSCs via CD63 receptors and induces the recruitment of intrahepatic neutrophils, ultimately leading to the formation of PMN [41]. A recent study revealed that cathepsin C derived from breast cancer promotes lung metastasis by modulating neutrophil infiltration and the formation of neutrophil extracellular traps. This finding suggests its potential utility as both a prognostic marker and a therapeutic target for breast cancer lung metastasis [24]. In the present study, elevated serum LBP levels in patients with GC-LM were firstly observed. Notably, GC patients with elevated serum LBP levels exhibited a heightened risk of postoperative GC-LM. These findings suggest the potential of LBP as a biomarker for the early detection of GC-LM and its critical role in PMN induction.

As indicated in previous studies, the accumulation of myeloid cells and deposition of ECM are recognized as pivotal characteristics of PMNs that influence the colonization of disseminated tumour cells in distant or secondary organs [30, 42, 43]. Here, it was observed that LBP promoted the colonization and initial outgrowth of GC cells in the liver at early stages in vivo. This finding suggests that LBP may play a role in reshaping myeloid cell accumulation and ECM deposition to promote GC-LM. As expected, the results confirmed that LBP induced the formation of fibrotic PMN by increasing the deposition of FN and COL1 in the liver. Consistently, Nielsen et al. demonstrated that granulin, secreted by metastasis-associated macrophages, can activate HSCs, leading to the development of a fibrotic microenvironment that promotes the LM of pancreatic ductal adenocarcinoma [44]. Furthermore, LBP pre-modeling led to the accumulation of myeloid cells in the liver in vivo*,* although the difference was not statistically significant. Interestingly, Jae W. Lee et al. also found that matrix deposition did not require myeloid cells [25]. Therefore, these results indicated that myeloid cells are not essential for ECM deposition in the liver.

HSCs are identified as the primary source of ECM in the liver [45], and are recognized as the central effectors of liver fibrosis [46]. However, the results of this study demonstrated that LBP mainly colocalized with intrahepatic macrophages in vitro and in vivo. A recent study highlighted the critical role of communication between KCs and HSCs in the development of liver fibrosis in alcohol-associated liver disease [47]. Therefore, it is proposed that intrahepatic macrophages mediate the formation of the fibrotic PMN through crosstalk with HSCs. Generally, secreted proteins execute their biological functions by binding to specific receptors on certain cell types, thereby initiating downstream signaling pathways. Using IP and Co-IP, here, it was found that LBP mainly interacts with the TLR4 receptor in intrahepatic macrophages. In line with data from the Human Protein Atlas Database, intrahepatic macrophages exhibited a high TLR4 expression, providing a partial explanation for the predominant accumulation of LBP in the liver and its colocalization with intrahepatic macrophages.

TGF-β is recognized as the pivotal cytokine driving fibrogenesis in the liver by inducing the activation of HSCs, which subsequently leads to ECM deposition [48]. González-Ramos et al. reported that extracellular HSP70 can bind to TLR4, inducing the expression of TGF-β1 in smooth muscle cells by enhancing the AP-1-dependent transcriptional activity. Likewise, Fu Jun Li et al. found that citrullinated vimentin can trigger the production of TGF-β1 through the activation of NF-κB in a TLR4-dependent manner, thereby promoting lung fibrosis [49]. Similarly, the results of the present study demonstrated that LBP promoted the secretion of TGF-β1 in intrahepatic macrophages by activating NF-κB in a TLR4-dependent manner. Consequently, TGF-β1 then activated HSCs, leading to an increased deposition of ECM in the liver. Moreover, TGF-β1 is recognized as a pivotal cytokine that plays a crucial role in promoting EMT, proliferation, stemness, immune evasion and metastasis across various cancer types [50, 51]. Nevertheless, systemic inhibition of TGF-β may pose challenges in clinical applications due to its involvement in multiple physiological homeostatic processes, potentially leading to side effects in patients [52]. Hence, small molecule compounds selectively targeting the TGF-β pathway have exhibited promising efficacy in both preclinical and clinical studies, serving as potential anti-tumour and anti-metastasis agents.

Galunisertib, a selective small molecule inhibitor of TGF-β receptor type I kinase tested in clinical trials, demonstrates a promising anti-tumour effect in rectal cancer, liver cancer, pancreatic cancer and various other solid tumours [53–56]. Notably, the in vivo results of the present study indicate that galunisertib is more effective than the anti-LBP antibody in preventing GC-LM. Interestingly, high expression of TGFBR1 were observed in GC, which correlated with disease progression and a worse prognosis in patients with GC. To this end, it is proposed that elevated intrahepatic levels of TGF-β1 could augment the migration and invasion capabilities of incoming disseminated GC cells, thereby further facilitating their colonization in the liver. Consistently, the results of the present study demonstrated that CM from THP-1 cells pretreated with reLBP (resulting in increased TGF-β1 levels) significantly enhanced the migration and invasion capabilities of GC cells through the upregulation of p-Smad3. Notably, galunisertib treatment extraordinarily attenuated these effects. However, further investigation is warranted to verify the effect of galunisertib on GC proliferation and its potential role in immune evasion in future studies. Taken together, the results of this study highlight the role of TGF-β1 in orchestrating the formation of fibrotic PMN initiated by GC-derived LBP. Moreover, intrahepatic TGF-β1 may further potentiate the migration and invasion of disseminated GC cells within the liver.

## Conclusion

**Fig. 8:**
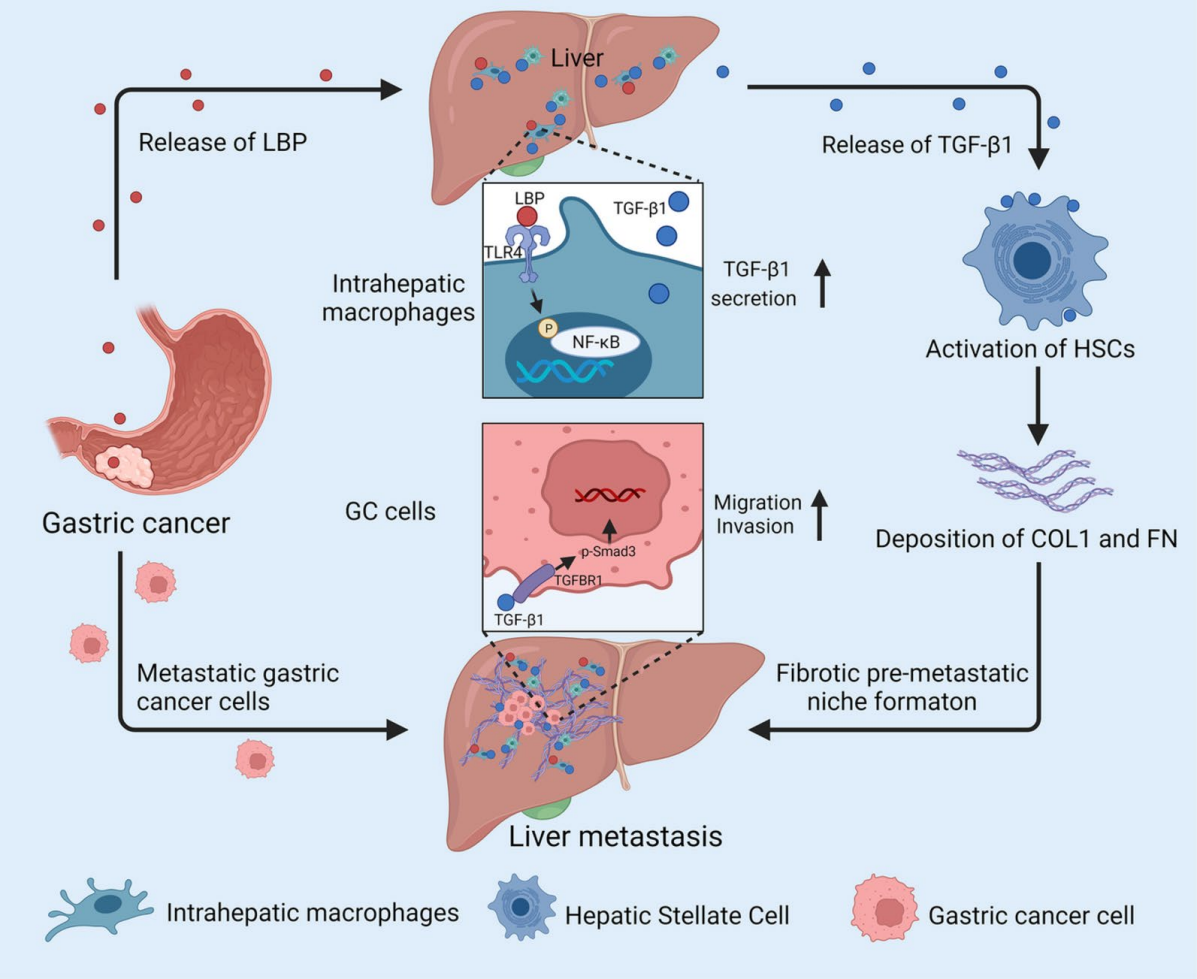
The schematic diagram of GC-derived LBP promoting LM. GC-derived LBP activates the TLR4/NF-κB pathway to promote TGF-β1 secretion in intrahepatic macrophages, in turn, TGF-β1 activates HSCs to direct intrahepatic fibrotic PMN. Additionally, intrahepatic TGF-β1 enhances the migration and invasion of metastatic GC cells in the liver

In summary, this study presented a novel role of GC-derived LBP in the context of GC-LM. Specifically, it was determined that GC-derived LBP activates the TLR4/NF-κB pathway in intrahepatic macrophages, leading to an elevated secretion of TGF-β1. Subsequently, TGF-β1 further activates HSCs, orchestrating the formation of an intrahepatic fibrotic PMN. Additionally, intrahepatic TGF-β1 enhances the migration and invasion of metastatic GC cells within the liver (Fig. 8). Importantly, these findings suggest that serum LBP could serve as a novel diagnostic biomarker for early detection of GC-LM. Furthermore, selective targeting of the TGF-β pathway with galunisertib has emerged as a promising strategy for both the prevention and treatment of GC-LM.

### Supplementary Information

**Additional file 1. **

**Additional file 2. **

**Additional file 3: Supplementary Table 3.** Primers and shRNAs used in this study.

**Additional file 4: Supplementary Table 4.** Antibodies used in this study.

**Additional file 5. **

**Additional file 6. **

**Additional file 7: Figure S1.** A. Venn diagram showed the promising proteins associated with GC-LM by overlapping proteins upregulated (log2FC > 1.0) in serum and mRNA upregulated (log2FC > 1.0) in GC tissues. B. The relative mRNA expression of LBP and RBP4 was determined by qRT-PCR in GC tissues and matched adjacent normal gastric tissues of 30 patients with stage II /III GC, and primary GC tissues of 20 GC-LM patients. C. Representative IHC images of LBP expression in normal gastric tissues and primary tumours of patients with GC at different stages. Scale bar, 200μm. D. Fold changes of LBP in GC tissues compared with normal tissues based on the relative expression detected by WB. E-F. The disease-free survival (E) and overall survival (F) of GC patients based on LBP expression in TCGA dataset. Data pooled as mean ± SEM of three biologically independent experiments, and p values were determined by one-way ANOVA test (B) or log rank test (E, F) (* *P* < 0.05, ** *P* < 0.01, *** *P* < 0.001). **Figure S2.** A. LBP mRNA levels were explored in normal gastric epithelial cell line GES-1 and seven GC cell lines by qRT-PCR. B-D. Validation of LBP stable overexpression in AGS cells and LBP stable knockdown in MKN45 cells by qRT-PCR (B, C) and WB (D), respectively. E. The modified procedures (left, 1 - 6) of intrasplenic injection mouse model of LM without tumour disseminated in abdominal cavity (right). F-G. Mice were euthanized in the 5th week after intrasplenic injection of AGS cells with or without LBP stable overexpression. Quantification of liver weight (F) and surface liver metastasis nodules (G) are shown. Data are representative of three independent experiments. Data are shown as mean ± SD, and p values were determined by one-way ANOVA test (A, C)，or two-tailed unpaired t test (B, F, G) (* *P* < 0.05, ** *P* < 0.01, *** *P* < 0.001). **Figure S3.** A. EdU and colony formation assays were applied to validate the effect of LBP on the proliferation of HGC27 cells with LBP stable overexpression or knockdown. B. Flow cytometry analysis showed apoptosis of HGC-27 cells with LBP stable overexpression or knockdown. C. Tumor sphere formation of MKN45 cells (top) and HGC-27 cells (bottom) with LBP stable overexpression or knockdown. Scale bars, 100μm. D. Quantification of the migration and invasion of MKN45 cells with LBP stable overexpression or knockdown are shown. E. The effect of LBP on the migration and invasion of HGC-27 cells was validated by wound-healing assays (row 1, 2) and Transwell assays (row 3, 4). F. Quantification of the migration and invasion of HGC-27 cells with LBP stable overexpression or knockdown are shown. G-H. Quantification of wound-healing assays showed no significance in MKN45 cells and HGC-27 cells with LBP stable overexpression or knockdown. I. WB was applied to confirm that LBP has no significant effect on proliferation, apoptosis and EMT of MKN45 cells. J. Quantification of representative IF images of EMT markers in MKN45 with LBP stable overexpression or knockdown by using Image J. Scale bars, 100μm (A, C, E). Data are representative of three independent experiments. Data are shown as mean ± SD of 3 biologically independent experiments, p values were determined by one-way ANOVA test (ns, not significant). **Figure S4.** A. Mice were euthanized in the 4th week after intrasplenic injection of AGS cells. Representative bioluminescent images (column of IVIS image), photographs (column of whole liver) and H&E staining with enlargement (columns of H&E and enlargement) of liver metastases are shown, respectively. B. Quantification of liver photon flux. C. Quantification of surface liver metastasis nodules. D. Representative bioluminescent images (left) and BLI analyses (right) of mice with intrasplenic injection of MKN45 cells (1× 10^6^ cells per mouse) after reLBP or IgG pre-modelling by tail vein injection (n=5 mice per group). E. Mice were euthanized in the 4th week after intrasplenic injection of MKN45 cells. Representative bioluminescent images (column of IVIS image), photographs (column of whole liver) and H&E staining with enlargement (columns of H&E and enlargement) of liver metastases are shown, respectively. F. Gating strategy for identification of CD45+, CD11b, Ly6G, and F4/80+ cells isolated from the liver of mouse with or without reLBP pre-modelling. G-H. Quantification of neutrophils (G) and macrophages (H) in the liver by fluorescence-activated cell sorting (FACS) analyses. I. Representative IHC images of α-SMA and COL1 in the livers of mice after PBS, IgG or reLBP pre-modelling. J. Representative IHC and IF images of CD31 in the livers after PBS, IgG or reLBP pre-modelling. K. The expression of ECM proteins and α-SMA was determined by WB in mouse liver tissues after PBS, IgG or reLBP pre-modelling. L. α-SMA levels of primary HSCs in different groups were determined by immunofluorescence staining. Scale bars are 1.0cm (A-whole liver, E-whole liver), 3mm (A- H&E, E- H&E), 300μm (A- enlargement, E- enlargement), 50μm (I, J- IHC), 100μm (J- IF) and 20μm (L), the colored scale bars represent the intensity of luminescence as indicated, respectively. Data are representative of three independent experiments. Data represent mean ± SD (B, C, G, H), or mean ± SEM (D), and p values were determined by two-tailed unpaired t test (B, C), one-way ANOVA test (G, H) or two-way ANOVA test (D) (* *P* < 0.05, ** *P* < 0.01, *** *P* < 0.001). **Figure S5.** A. Study design for investigating the accumulation of reLBP-GFP protein in mice after tail vein injection* in vivo*. B. Quantification of the fluorescence intensity of liver, lung, bone, kidney and spleen tissues with tail injection of reLBP-GFP at the 24th hour. C. Representative H&E and IF images of accumulation of reLBP-GFP protein in mouse tissue sections after tail vein injection. D-E. Changes of morphology (D) and CD68 expression (E) were detected by microscopy and IF in THP-1 cells after stimulation with phorbol 12-myristate 13-acetate (PMA) for 72 hours. F. Study design for macrophage depletion with CEL to validate the effect of macrophage on the fibrotic microenvironment induced by reLBP in mouse liver. G. Representative IF images presented that reLBP-GFP colocalized with PMA treated THP-1 *in vitro*. Data are representative of three independent experiments. Scale bars are 200μm (C- H&E), 20μm (C- IF, D, E, G). Data are shown as mean ± SD, and p values were determined by one-way ANOVA test (B) (* *P* < 0.05, ** *P* < 0.01, *** *P* < 0.001). **Figure S6.** A. Prediction of proteins interacted with LBP from the STRING databases (https://string-db.org/). B. Representative secondary mass spectrum of LBP protein. C. Protein-protein docking analysis of LBP and TLR4 in the HDOCK SERVER database (http://hdock.phys.hust.edu.cn/). D. TLR4 expression in RNA single cell types in the Human Protein Atlas database showed that TLR4 is enriched in KCs. E. KEGG analysis indicated functional targets of LBP in macrophages based on mRNA sequencing in PMA-treated THP-1 cells with IgG or reLBP pretreatment. F. WB was performed to investigate the regulatory mechanism of LBP in THP1 *in vitro*. Data are representative of three independent experiments. **Figure S7.** A. Representative H&E and IF images showed the anti-LBP antibody and galunisertib blocked the pro-fibrotic effect of reLBP in mouse liver *in vivo*. B. Quantitation of liver metastasis nodules in mice with intrasplenic injection of MKN45 cells (1 × 10^6^ cells per mouse) showed the inhibitory effect of the anti-LBP antibody and galunisertib on LM* in vivo*. (n = 5 per group). C-D. The expression of TGFBR1 in GC tissues vs normal tissues (C), and in GC tissues with different stages (D) are shown based on TCGA database. E. The protein expression levels of TGFBR1 in multiple cancer types in the Human Protein Atlas database. F. Representative IF images of TGFBR1 expression in GES-1 and GC cell lines (AGS, HGC-27, MKN45). G. The overall survival of patients with GC based on TGFBR1 expression in TCGA database. H. Quantitation of the formation of filopodium-like protrusions (FLPs) per cell in MKN45 cells treated with the CM from reLBP-treated THP-1 or galunisertib. Scale bars are 200μm (A-H&E), 100μm (A-IF), 20μm (F). Data are representative of three independent experiments. Data are shown as mean ± SEM (B), or mean ± SD (C), and p values were determined by one-way ANOVA test (B), two-tailed unpaired t test (C), or log rank test (G) (* *P* < 0.05, ** *P* < 0.01, *** *P* < 0.001).

## Data Availability

The raw mRNA-seq data, as well as all other data supporting the results of this study, are available from the corresponding author upon reasonable request.

## Abbreviations

GC: Gastric cancer
LM: Liver metastasis
GC-LM: Liver metastasis of gastric cancer
PMN: Pre-metastatic niche
LBP: Lipopolysaccharide binding protein
HSCs: Hepatic satellite cells
mRNA-seq: mRNA sequencing
IVIS: In vivo Imaging system
PMA: Phorbol 12-myristate 13-acetate
ECM: Extracellular matrix
CEL: Clodronate-encapsulated liposomes
CM: Conditioned medium
IHC: Immunohistochemistry
IF: Immunofluorescence
