# Supplementary material for: Gastric cancer-derived LBP promotes liver metastasis by driving intrahepatic fibrotic pre-metastatic niche formation
Source: J Exp Clin Cancer Res. 2023 Oct 3;42:258. doi: 10.1186/s13046-023-02833-8 (PMC10546721; doi:10.1186/s13046-023-02833-8)
Supplement: Supplementary file 3 — Additional file 3: Supplementary Table 3. Primers and shRNAs used in this study. [file 13046_2023_2833_MOESM3_ESM.docx]

**Supplementary Table 3**

**Primers and shRNAs used in this study**

| **Gene name** | **Sequence (5' - 3')** |
| --- | --- |
| qRT-PCR primers |  |
| LBP | F: CTACAGGGCTCCTTTGATGTCA |
|  | R: CACGTCAGCGATGTCACTG |
| RBP4 | F: GCCTCTTTCTGCAGGACAAC |
|  | R: GCACACGTCCCAGTTATTCA |
| GAPDH | F: CGGAGTCAACGGATTTGGTCGTAT |
|  | R: AGCCTTCTCCATGGTGGTGAAGAC |
| ACTB | F: CATGTACGTTGCTATCCAGGC |
|  | R: CTCCTTAATGTCACGCACGAT |
| TGFB1 | F: GGCCAGATCCTGTCCAAGC |
|  | R: GTGGGTTTCCACCATTAGCAC |
| shRNAs |  |
| shLBP-1 | CCGGGCCGACATTGATTATAGCTTACTCGAGTAAGCTATAATCAATGTCGGCTTTTTT |
| shLBP-2 | CCGGCCTCAACTATTACATCCTTAACTCGAGTTAAGGATGTAATAGTTGAGGTTTTTT |
