# Supplementary material for: Gastric cancer-derived LBP promotes liver metastasis by driving intrahepatic fibrotic pre-metastatic niche formation
Source: J Exp Clin Cancer Res. 2023 Oct 3;42:258. doi: 10.1186/s13046-023-02833-8 (PMC10546721; doi:10.1186/s13046-023-02833-8)
Supplement: Supplementary file 4 — Additional file 4: Supplementary Table 4. Antibodies used in this study. [file 13046_2023_2833_MOESM4_ESM.docx]

**Supplementary Table 4**

**Antibodies used in this study**

| **Antibodies** | **Source** | **Identifier** |
| --- | --- | --- |
| Albumin (for ICC/IF 1:500) | CST | ab207327 |
| CD11b (for FACS analyses) | Biolegend | 101206 |
| CD31 (for IHC/IF 1:200) | Abcam | ab281583 |
| CD45 (for FACS analyses) | Biolegend | 147712 |
| CD68 (for ICC/IF 1:200) | eBioscience | 14-0688-82 |
| Cleaved-caspace-3 (for WB 1:1000) | CST | 9664T |
| Collagen I (for WB 1:1000, for ICC/IF 1:200) | CST | 72026S |
| Desmin (for ICC/IF 1:400) | Proteintech | 16520-1-AP |
| E-cadherin (for WB 1:1000, for ICC/IF 1:200) | CST | 14472S |
| F4/80 (for FACS analyses) | eBioscience | 17-4801-82 |
| F4/80 (for ICC/IF 1:200) | CST | 70076S |
| Fibronectin (for ICC 1:400) | eBioscience | 14-9869-82 |
| Fibronectin (for WB 1:1000, for IHC/IF 1:200) | Abcam | ab2413 |
| GAPDH (for WB 1:1000) | CST | 5154S |
| Ki-67 (for IHC 1:500) | CST | 34330SF |
| LBP (for IHC/IF 1:200) | R&D | AF870 |
| LBP (for WB 1:1000) | Abcam | ab169776 |
| LBP (Neutralizing *in vivo*, 200ug/mouse) | R&D-Biotechne | MAB6635 |
| Ly6G (for IF 1:100) | eBioscience | 14-5931-82 |
| Ly6G (for FACS analyses) | eBioscience | 11-9668-82 |
| N-cadherin (for WB 1:1000) | CST | 13116S |
| NF-κB p65 (for WB 1:1000) | CST | 8242T |
| p-ERK1/2 (for WB 1:1000) | CST | 9101S |
| p-NF-κB p65(Ser536) (for WB 1:1000, for ICC/IF:1:200) | CST | 3033S |
| p-Smad3 (for WB 1:2000, for ICC/IF 1:250) | Abcam | ab52903 |
| Tenasin C (for WB 1:2000) | Abcam | ab108930 |
| TGFBR1 (for IHC 1:1000, for IF 1:500) | Invitrogen | PA5-98192 |
| TGF-β (Neutralizing *in vitr*o, 50ug/ml) | Bioxcell | BP0057 |
| TGF-β1 (for ICC/IF 1:200) | Invitrogen | MA1-21595 |
| TGF-β1 (for WB 1:1000) | Abcam | ab215715 |
| TLR4 (for ICC 1:200) | Invitrogen | PA5-142481 |
| TLR4 (for WB 1:1000) | Proteintech | 19811-1-AP |
| Vimentin (for WB 1:1000, for ICC/IF 1:200) | CST | 5741T |
| Vitronectin (for WB 1:2000) | Abcam | ab272865 |
| α-SMA (for ICC 1:300) | NOVAS | NB300-978 |
| α-SMA (for WB 1:1000, for IF 1:400) | CST | 19245S |
| β-Actin (for WB 1:1000) | CST | 4970S |
| Anti-mouse IgG HRP-linked Antibody (for WB 1:3000) | CST | 7076S |
| Anti-rabbit IgG HRP-linked Antibody (for WB 1:3000) | CST | 7074S |
| Donkey Anti-Goat IgG H&L (Alexa Fluor 488) (for IF 1:200) | Abcam | ab150129 |
| Donkey Anti-Goat IgG H&L (Alexa Fluor 555) (for IF 1:200) | Abcam | ab150130 |
| Donkey Anti-Mouse IgG H&L (Alexa Fluor 488) (for IF 1:200) | Abcam | ab150105 |
| Donkey Anti-Mouse IgG H&L (Alexa Fluor 555) (for IF 1:200) | Abcam | ab150106 |
| Donkey Anti-Mouse IgG H&L (Alexa Fluor 647) (for IF 1:200) | Abcam | ab150107 |
| Donkey Anti-Rat IgG H&L (Alexa Fluor 594) (for IF 1:200) | Abcam | ab150156 |
| Donkey Anti-Rbbit IgG H&L (Alexa Fluor 488) (for IF 1:200) | Abcam | ab150073 |
| Donkey Anti-Rbbit IgG H&L (Alexa Fluor 555) (for IF 1:200) | Abcam | ab150074 |
