# Supplementary material for: Gastric cancer-derived LBP promotes liver metastasis by driving intrahepatic fibrotic pre-metastatic niche formation
Source: J Exp Clin Cancer Res. 2023 Oct 3;42:258. doi: 10.1186/s13046-023-02833-8 (PMC10546721; doi:10.1186/s13046-023-02833-8)
Supplement: Supplementary file 7 — Additional file 7: Figure S1. A. Venn diagram showed the promising proteins associated with GC-LM by overlapping proteins upregulated (log2FC > 1.0) in serum and mRNA upregulated (log2FC > 1.0) in GC tissues. B. The relative mRNA expression of LBP and RBP4 was determined by qRT-PCR in GC tissues and matched adjacent normal gastric tissues of 30 patients with stage II /III GC, and primary GC tissues of 20 GC-LM patients. C. Representative IHC images of LBP expression in normal gastric tissues and primary tumours of patients with GC at different stages. Scale bar, 200μm. D. Fold changes of LBP in GC tissues compared with normal tissues based on the relative expression detected by WB. E-F. The disease-free survival (E) and overall survival (F) of GC patients based on LBP expression in TCGA dataset. Data pooled as mean ± SEM of three biologically independent experiments, and p values were determined by one-way ANOVA test (B) or log rank test (E, F) (* P < 0.05, ** P < 0.01, *** P < 0.001). Figure S2. A. LBP mRNA levels were explored in normal gastric epithelial cell line GES-1 and seven GC cell lines by qRT-PCR. B-D. Validation of LBP stable overexpression in AGS cells and LBP stable knockdown in MKN45 cells by qRT-PCR (B, C) and WB (D), respectively. E. The modified procedures (left, 1 - 6) of intrasplenic injection mouse model of LM without tumour disseminated in abdominal cavity (right). F-G. Mice were euthanized in the 5th week after intrasplenic injection of AGS cells with or without LBP stable overexpression. Quantification of liver weight (F) and surface liver metastasis nodules (G) are shown. Data are representative of three independent experiments. Data are shown as mean ± SD, and p values were determined by one-way ANOVA test (A, C)，or two-tailed unpaired t test (B, F, G) (* P < 0.05, ** P < 0.01, *** P < 0.001). Figure S3. A. EdU and colony formation assays were applied to validate the effect of LBP on the proliferation of HGC27 cells with LBP stable overexpr [file 13046_2023_2833_MOESM7_ESM.docx]

**Supplementary Figure 1**

**
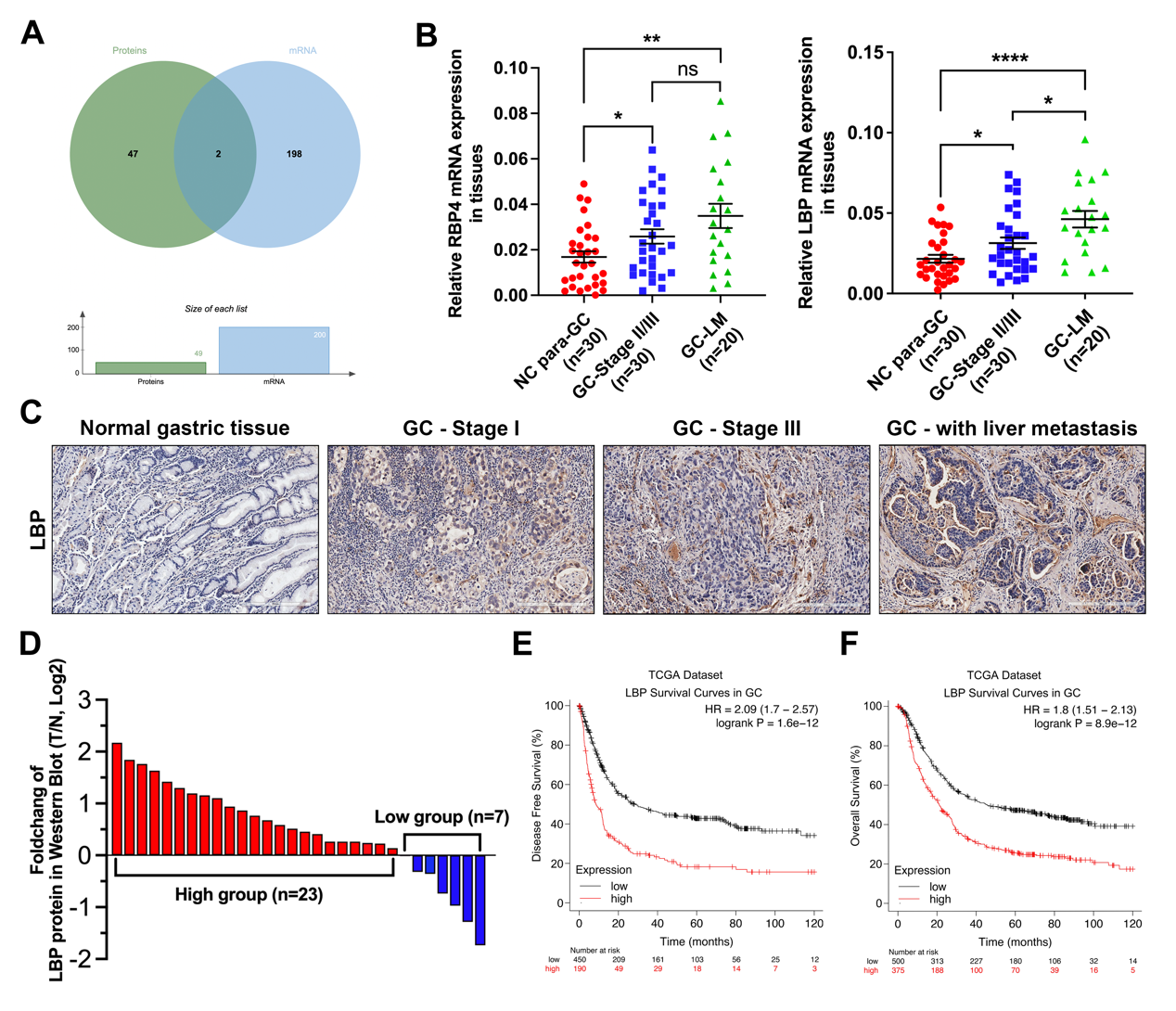
**

**Figure S1. A.** Venn diagram showed the promising proteins associated with GC-LM by overlapping proteins upregulated (log2FC > 1.0) in serum and mRNA upregulated (log2FC > 1.0) in GC tissues. **B**. The relative mRNA expression of LBP and RBP4 was determined by qRT-PCR in GC tissues and matched adjacent normal gastric tissues of 30 patients with stage II /III GC, and primary GC tissues of 20 GC-LM patients. **C**. Representative IHC images of LBP expression in normal gastric tissues and primary tumours of patients with GC at different stages. Scale bar, 200μm. **D**. Fold changes of LBP in GC tissues compared with normal tissues based on the relative expression detected by WB. **E-F**. The disease-free survival (**E**) and overall survival (**F**) of GC patients based on LBP expression in TCGA dataset. Data pooled as mean ± SEM of three biologically independent experiments, and p values were determined by one-way ANOVA test (**B**) or log rank test (**E**, **F**) (* P < 0.05, ** P < 0.01, *** P < 0.001).

**Supplementary Figure 2**

**
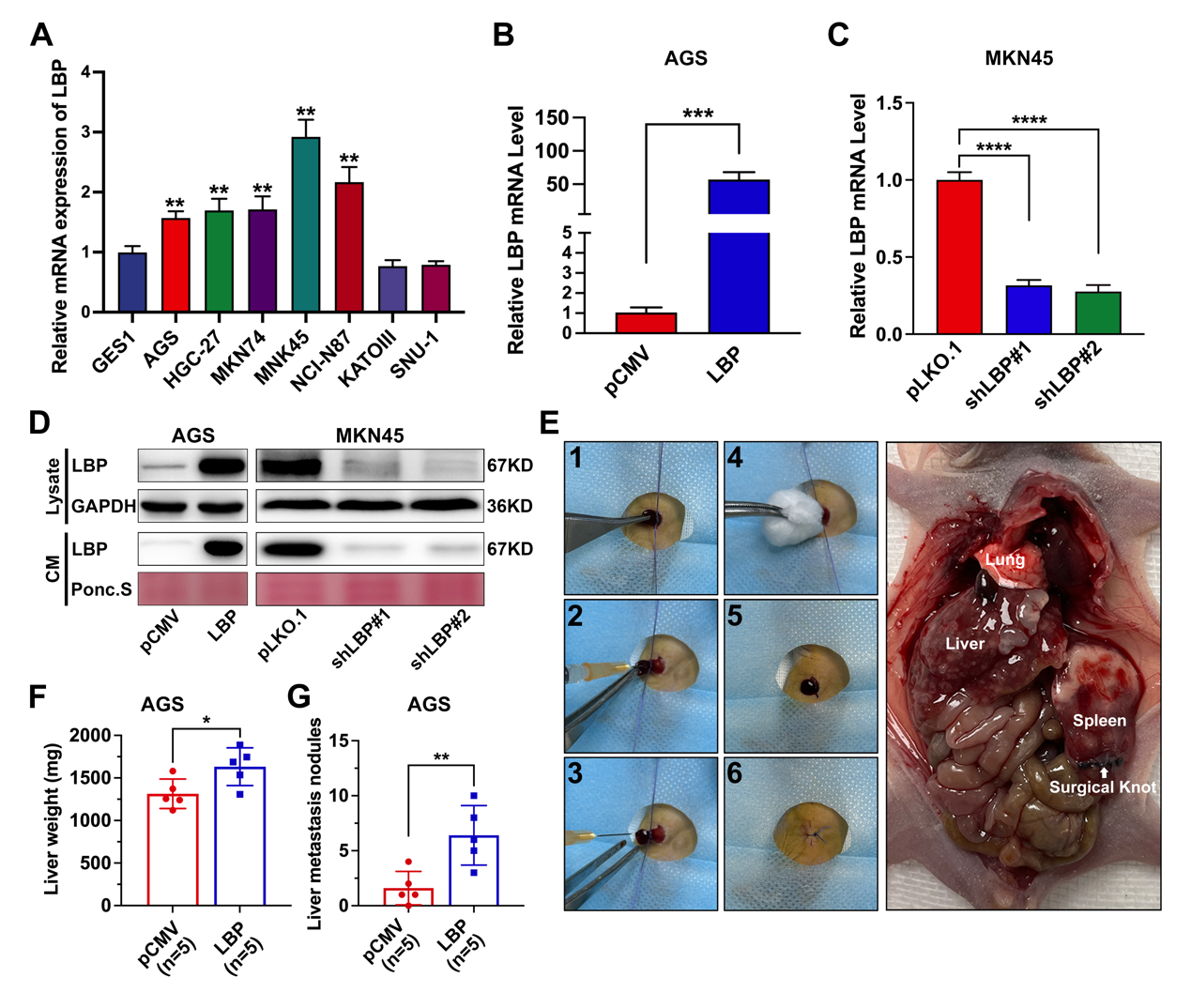
**

**Figure S2. A.** LBP mRNA levels were explored in normal gastric epithelial cell line GES-1 and seven GC cell lines by qRT-PCR. **B-D.** Validation of LBP stable overexpression in AGS cells and LBP stable knockdown in MKN45 cells by qRT-PCR (**B**, **C**) and WB (**D**), respectively. **E**. The modified procedures (left, 1 - 6) of intrasplenic injection mouse model of LM without tumour disseminated in abdominal cavity (right). **F-G**. Mice were euthanized in the 5th week after intrasplenic injection of AGS cells with or without LBP stable overexpression. Quantification of liver weight (**F**) and surface liver metastasis nodules (**G**) are shown. Data are representative of three independent experiments. Data are shown as mean ± SD, and p values were determined by one-way ANOVA test (**A**, **C**)，or two-tailed unpaired t test (**B**, **F**, **G**) (* P < 0.05, ** P < 0.01, *** P < 0.001).

**Supplementary Figure 3**


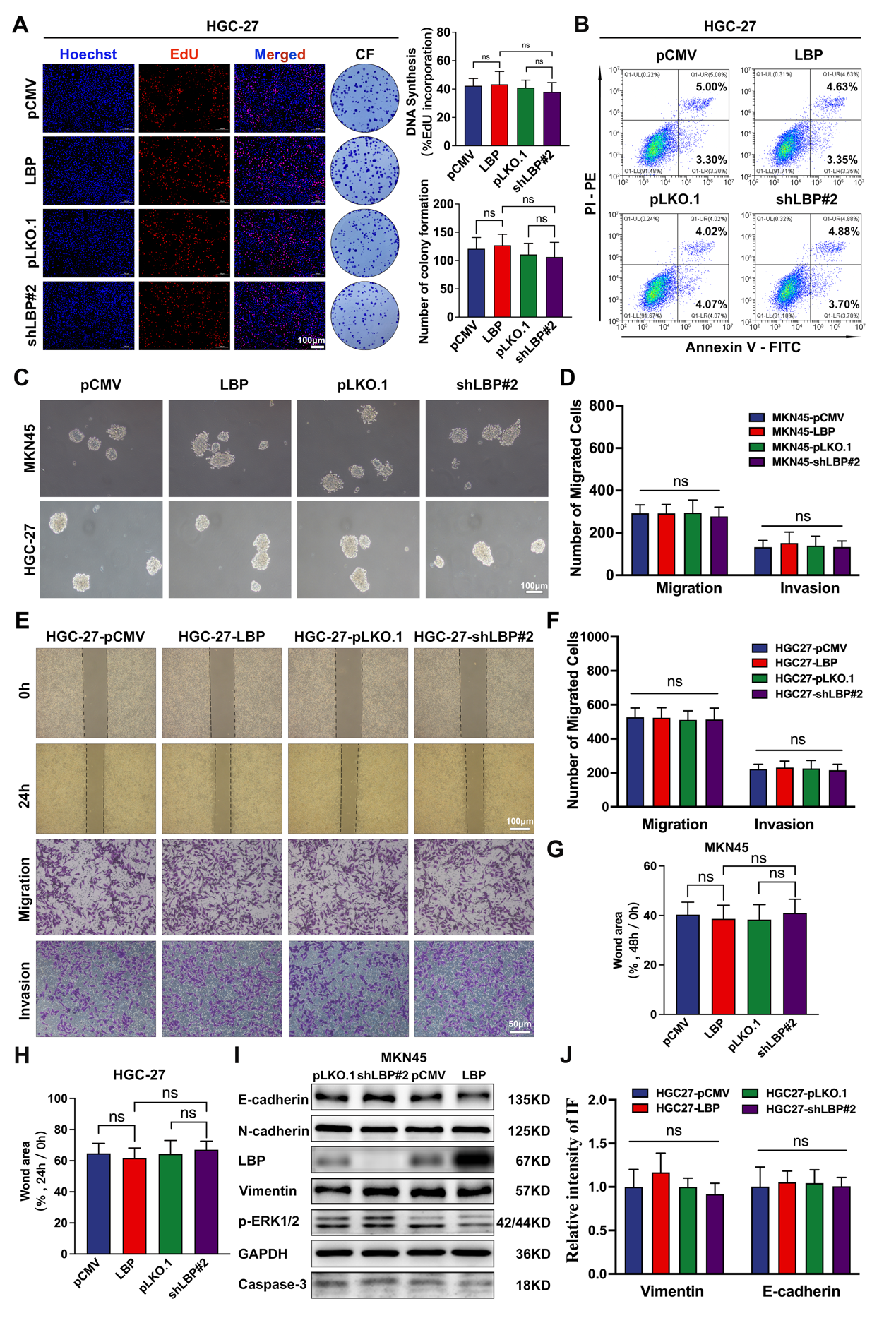


**Figure S3. A**. EdU and colony formation assays were applied to validate the effect of LBP on the proliferation of HGC27 cells with LBP stable overexpression or knockdown. **B**. Flow cytometry analysis showed apoptosis of HGC-27 cells with LBP stable overexpression or knockdown. **C**. Tumor sphere formation of MKN45 cells (top) and HGC-27 cells (bottom) with LBP stable overexpression or knockdown. Scale bars, 100μm. **D**. Quantification of the migration and invasion of MKN45 cells with LBP stable overexpression or knockdown are shown. **E.** The effect of LBP on the migration and invasion of HGC-27 cells was validated by wound-healing assays (row 1, 2) and Transwell assays (row 3, 4). **F**. Quantification of the migration and invasion of HGC-27 cells with LBP stable overexpression or knockdown are shown. **G-H.** Quantification of wound-healing assays showed no significance in MKN45 cells and HGC-27 cells with LBP stable overexpression or knockdown. **I**. WB was applied to confirm that LBP has no significant effect on proliferation, apoptosis and EMT of MKN45 cells. **J**. Quantification of representative IF images of EMT markers in MKN45 with LBP stable overexpression or knockdown by using Image J. Scale bars, 100μm (**A**, **C, E**). Data are representative of three independent experiments. Data are shown as mean ± SD of 3 biologically independent experiments, p values were determined by one-way ANOVA test (ns, not significant).

**Supplementary Figure 4**


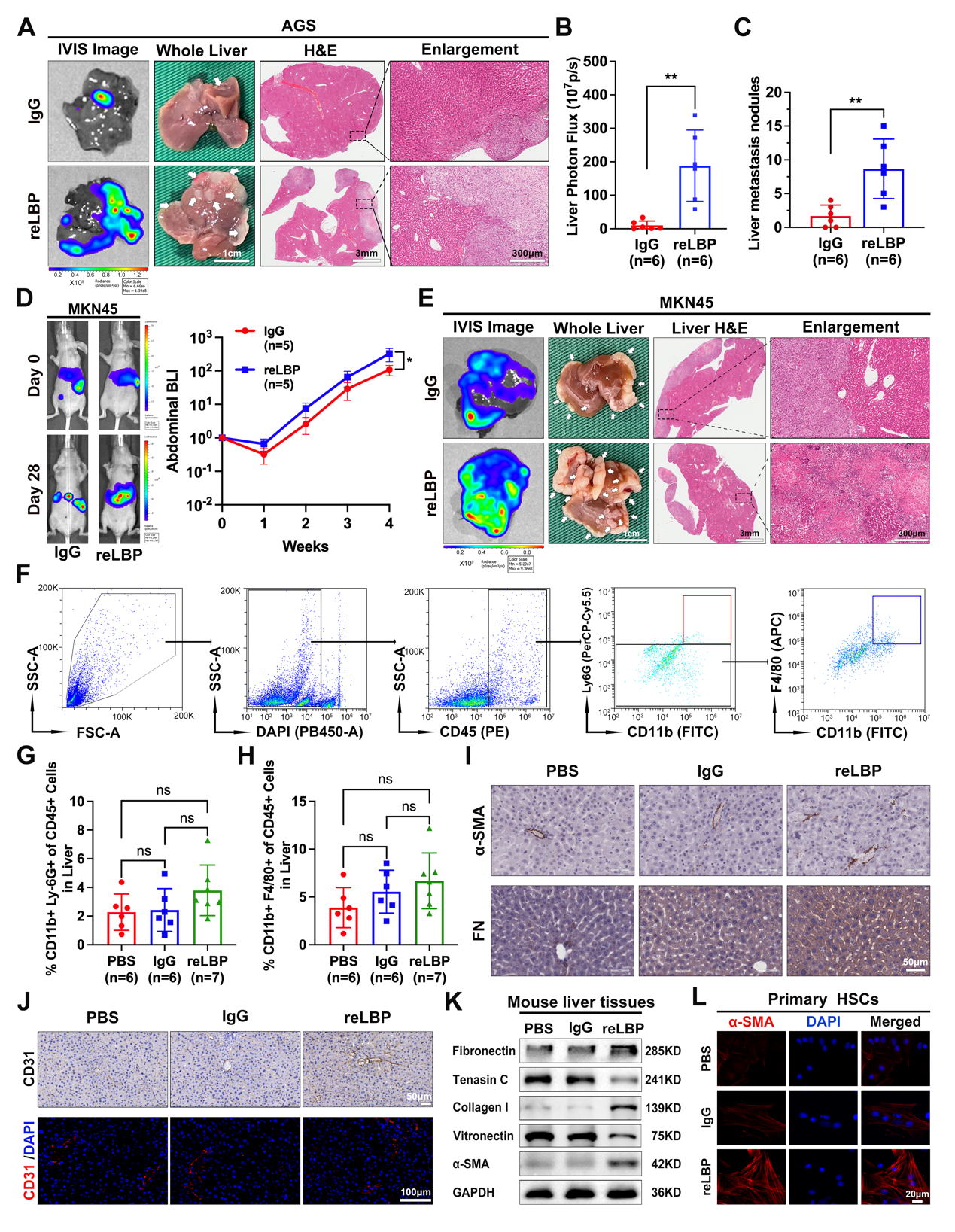


**Figure S4. A**. Mice were euthanized in the 4th week after intrasplenic injection of AGS cells. Representative bioluminescent images (column of IVIS image), photographs (column of whole liver) and H&E staining with enlargement (columns of H&E and enlargement) of liver metastases are shown, respectively. **B**. Quantification of liver photon flux. **C**. Quantification of surface liver metastasis nodules. **D**. Representative bioluminescent images (left) and BLI analyses (right) of mice with intrasplenic injection of MKN45 cells (1× 10^6^ cells per mouse) after reLBP or IgG pre-modelling by tail vein injection (n=5 mice per group). **E**. Mice were euthanized in the 4th week after intrasplenic injection of MKN45 cells. Representative bioluminescent images (column of IVIS image), photographs (column of whole liver) and H&E staining with enlargement (columns of H&E and enlargement) of liver metastases are shown, respectively. **F**. Gating strategy for identification of CD45+, CD11b, Ly6G, and F4/80+ cells isolated from the liver of mouse with or without reLBP pre-modelling. **G-H**. Quantification of neutrophils (**G**) and macrophages (**H**) in the liver by fluorescence-activated cell sorting (FACS) analyses. **I**. Representative IHC images of α-SMA and COL1 in the livers of mice after PBS, IgG or reLBP pre-modelling. **J**. Representative IHC and IF images of CD31 in the livers after PBS, IgG or reLBP pre-modelling. **K**. The expression of ECM proteins and α-SMA was determined by WB in mouse liver tissues after PBS, IgG or reLBP pre-modelling. **L**. α-SMA levels of primary HSCs in different groups were determined by immunofluorescence staining. Scale bars are 1.0cm (**A**-whole liver, **E**-whole liver), 3mm (**A**- H&E, **E**- H&E), 300μm (**A**- enlargement, **E**- enlargement), 50μm (**I**, **J**- IHC), 100μm (**J**- IF) and 20μm (**L**), the colored scale bars represent the intensity of luminescence as indicated, respectively. Data are representative of three independent experiments. Data represent mean ± SD (**B**, **C**, **G**, **H**), or mean ± SEM (**D**), and p values were determined by two-tailed unpaired t test (**B**, **C**), one-way ANOVA test (**G**, **H**) or two-way ANOVA test (**D**) (* P < 0.05, ** P < 0.01, *** P < 0.001).

**Supplementary Figure 5**

**
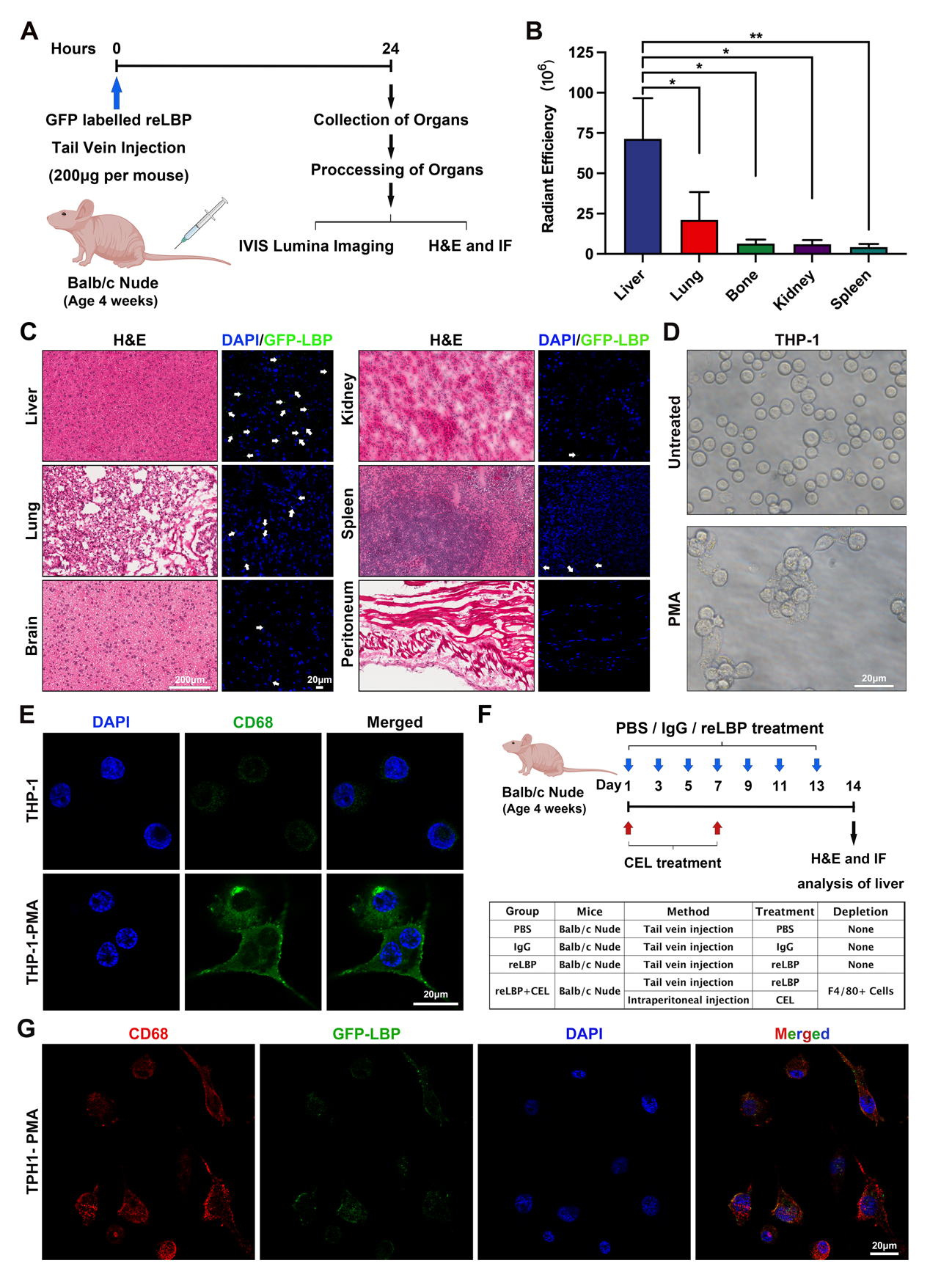
**

**Figure S5. A**. Study design for investigating the accumulation of reLBP-GFP protein in mice after tail vein injection *in vivo*. **B**. Quantification of the fluorescence intensity of liver, lung, bone, kidney and spleen tissues with tail injection of reLBP-GFP at the 24th hour. **C**. Representative H&E and IF images of accumulation of reLBP-GFP protein in mouse tissue sections after tail vein injection. **D-E**. Changes of morphology (**D**) and CD68 expression (**E**) were detected by microscopy and IF in THP-1 cells after stimulation with phorbol 12-myristate 13-acetate (PMA) for 72 hours. **F**. Study design for macrophage depletion with CEL to validate the effect of macrophage on the fibrotic microenvironment induced by reLBP in mouse liver. **G**. Representative IF images presented that reLBP-GFP colocalized with PMA treated THP-1 *in vitro*. Data are representative of three independent experiments. Scale bars are 200μm (**C**- H&E), 20μm (**C**- IF, **D**, **E**, **G**). Data are shown as mean ± SD, and p values were determined by one-way ANOVA test (**B**) (* P < 0.05, ** P < 0.01, *** P < 0.001).

**Supplementary Figure 6**


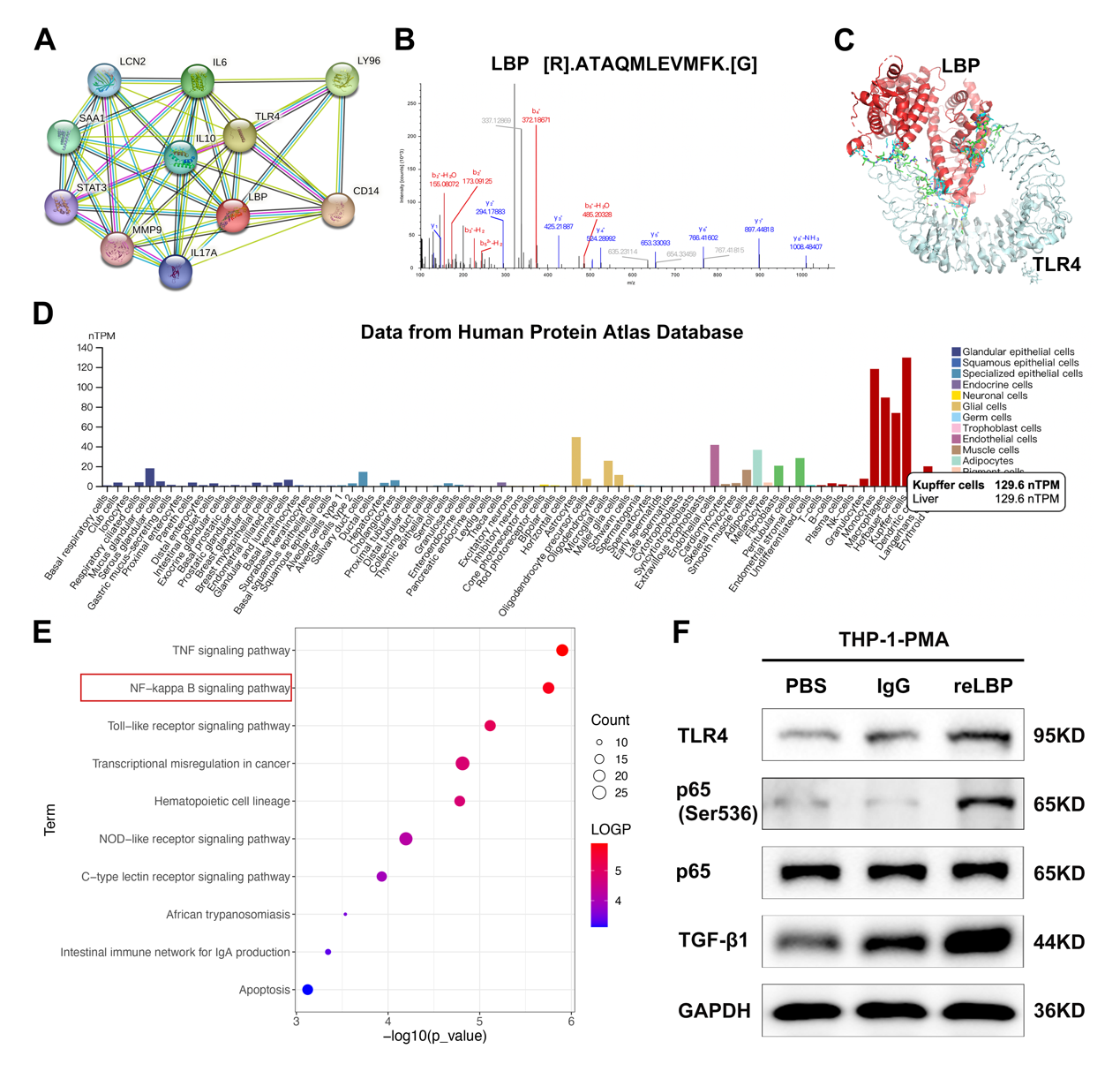


**Figure S6. A**. Prediction of proteins interacted with LBP from the STRING databases (<https://string-db.org/>). **B**. Representative secondary mass spectrum of LBP protein. **C**. Protein-protein docking analysis of LBP and TLR4 in the HDOCK SERVER database (http://hdock.phys.hust.edu.cn/). **D**. TLR4 expression in RNA single cell types in the Human Protein Atlas database showed that TLR4 is enriched in KCs. **E**. KEGG analysis indicated functional targets of LBP in macrophages based on mRNA sequencing in PMA-treated THP-1 cells with IgG or reLBP pretreatment. **F**. WB was performed to investigate the regulatory mechanism of LBP in THP1 *in vitro*. Data are representative of three independent experiments.

**Supplementary Figure 7**


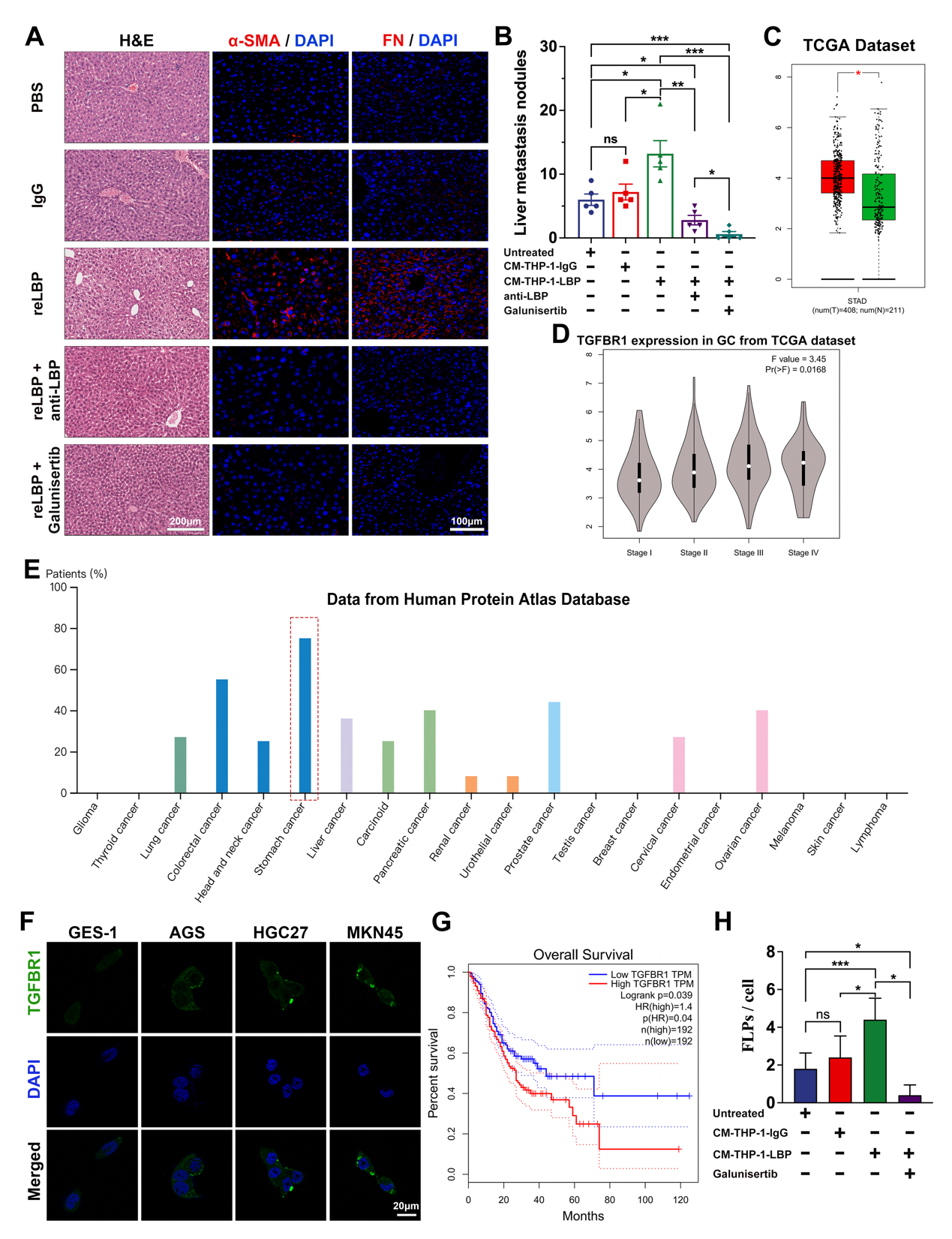


**Figure S7. A**. Representative H&E and IF images showed the anti-LBP antibody and galunisertib blocked the pro-fibrotic effect of reLBP in mouse liver *in vivo*. **B**. Quantitation of liver metastasis nodules in mice with intrasplenic injection of MKN45 cells (1 × 10^6^ cells per mouse) showed the inhibitory effect of the anti-LBP antibody and galunisertib on LM *in vivo*. (n = 5 per group). **C-D**. The expression of TGFBR1 in GC tissues vs normal tissues (**C**), and in GC tissues with different stages (**D**) are shown based on TCGA database. **E**. The protein expression levels of TGFBR1 in multiple cancer types in the Human Protein Atlas database. **F**. Representative IF images of TGFBR1 expression in GES-1 and GC cell lines (AGS, HGC-27, MKN45). **G.** The overall survival of patients with GC based on TGFBR1 expression in TCGA database. **H**. Quantitation of the formation of filopodium-like protrusions (FLPs) per cell in MKN45 cells treated with the CM from reLBP-treated THP-1 or galunisertib. Scale bars are 200μm (**A**-**H&E**), 100μm (**A-IF**), 20μm (**F**). Data are representative of three independent experiments. Data are shown as mean ± SEM (**B**), or mean ± SD (**C**), and p values were determined by one-way ANOVA test (**B**), two-tailed unpaired t test (**C**), or log rank test (**G**) (* P < 0.05, ** P < 0.01, *** P < 0.001).
